# NSUN2/YBX1 promotes the progression of breast cancer by enhancing HGH1 mRNA stability through m^5^C methylation

**DOI:** 10.1186/s13058-024-01847-0

**Published:** 2024-06-06

**Authors:** Xuran Zhang, Ke An, Xin Ge, Yuanyuan Sun, Jingyao Wei, Weihong Ren, Han Wang, Yueqin Wang, Yue Du, Lulu He, Ouwen Li, Shaoxuan Zhou, Yong Shi, Tong Ren, Yun-gui Yang, Quancheng Kan, Xin Tian

**Affiliations:** 1 Department of Pharmacy, The First Affiliated Hospital of Zhengzhou University, No.1 Jianshedong Rd, Zhengzhou, Henan 450052 China; 2 Henan Key Laboratory of Precision Clinical Pharmacy, Zhengzhou University, Zhengzhou, Henan 450052 China; 3 Department of Breast Surgery, The First Affiliated Hospital of Zhengzhou University, Zhengzhou, Henan 450052 China; 4 Department of Translational Medicine Center, The First Affiliated Hospital of Zhengzhou University, Zhengzhou, Henan 450052 China; 5 Department of Laboratory Medicine, The First Affiliated Hospital of Henan University of Chinese Medicine, Zhengzhou, Henan 450000 China; 6 Biobank of the First Affiliated Hospital of Zhengzhou University, Zhengzhou, Henan 450052 China; 7 China National Center for Bioinformation, Beijing, 100101 China; 8 University of Chinese Academy of Sciences, Beijing, 101408 China

**Keywords:** RNA 5-methylcytosinine, NSUN2, HGH1, Translation efficiency, Breast cancer

## Abstract

**Background:**

RNA m^5^C methylation has been extensively implicated in the occurrence and development of tumors. As the main methyltransferase, NSUN2 plays a crucial regulatory role across diverse tumor types. However, the precise impact of NSUN2-mediated m^5^C modification on breast cancer (BC) remains unclear. Our study aims to elucidate the molecular mechanism underlying how NSUN2 regulates the target gene *HGH1* (also known as *FAM203*) through m^5^C modification, thereby promoting BC progression. Additionally, this study targets at preliminarily clarifying the biological roles of NSUN2 and HGH1 in BC.

**Methods:**

Tumor and adjacent tissues from 5 BC patients were collected, and the m^5^C modification target *HGH1* in BC was screened through RNA sequencing (RNA-seq) and single-base resolution m^5^C methylation sequencing (RNA-BisSeq). Methylation RNA immunoprecipitation-qPCR (MeRIP-qPCR) and RNA-binding protein immunoprecipitation-qPCR (RIP-qPCR) confirmed that the methylation molecules NSUN2 and YBX1 specifically recognized and bound to *HGH1* through m^5^C modification. In addition, proteomics, co-immunoprecipitation (co-IP), and Ribosome sequencing (Ribo-Seq) were used to explore the biological role of HGH1 in BC.

**Results:**

As the main m^5^C methylation molecule, NSUN2 is abnormally overexpressed in BC and increases the overall level of RNA m^5^C. Knocking down NSUN2 can inhibit BC progression in vitro or in vivo. Combined RNA-seq and RNA-BisSeq analysis identified *HGH1* as a potential target of abnormal m^5^C modifications. We clarified the mechanism by which NSUN2 regulates HGH1 expression through m^5^C modification, a process that involves interactions with the YBX1 protein, which collectively impacts mRNA stability and protein synthesis. Furthermore, this study is the first to reveal the binding interaction between HGH1 and the translation elongation factor EEF2, providing a comprehensive understanding of its ability to regulate transcript translation efficiency and protein synthesis in BC cells.

**Conclusions:**

This study preliminarily clarifies the regulatory role of the NSUN2-YBX1-m^5^C-HGH1 axis from post-transcriptional modification to protein translation, revealing the key role of abnormal RNA m^5^C modification in BC and suggesting that HGH1 may be a new epigenetic biomarker and potential therapeutic target for BC.

**Supplementary Information:**

The online version contains supplementary material available at 10.1186/s13058-024-01847-0.

## Introduction

The incidence of breast cancer (BC) has increased, and it has become the leading malignant tumor in women. This situation emphasizes the importance of effective analysis strategies for early diagnosis and the search for therapeutic targets [1]. Currently, the clinical diagnosis of BC mainly relies on conventional histopathology and imaging, such as mammography, magnetic resonance imaging, and ultrasound. However, these methods exhibit certain limitations in early diagnosis as well as in detecting recurrence and metastasis [2]. Although well-established, treatments such as surgery, chemotherapy, adjunctive therapy, and targeted therapy are not effective for all types of BC [3]. Despite the maturity and application of these methods, complete eradication of the disease remains elusive. Recent advances in immunotherapy have been applied to BC, but they have yielded effective therapeutic effects in only a small subset of patients [4–8]. Therefore, the clinical treatment of BC still faces significant challenges, necessitating the urgent exploration of new mechanisms, biomarkers, and treatment targets.

The field of epigenetics has made significant strides in elucidating the mechanisms of complex diseases, particularly tumors [9–11]. As an epigenetic regulatory mechanism, RNA m^5^C methylation has been widely identified in eukaryotes and is associated with cell differentiation, development, and tumor progression [12–16]. NSUN2, a key methylation molecule in RNA m^5^C modification, acts as a methyltransferase that transfers methyl groups to cytosine residues on tRNAs, mRNAs, and noncoding RNAs, thus serving as an m^5^C “writer” [17–20]. Many studies have demonstrated that elevated NSUN2 expression enhances the m^5^C level of transcripts in tumors. Through miCLIP-Seq, MeRIP-Seq, or RNA-BisSeq, modified target genes of m^5^C in tumors have been identified, confirming that NSUN2 regulates these target genes through m^5^C modification, thereby exerting oncogenic effects. For instance, high NSUN2 expression in esophageal cancer correlates with poor patient survival, and its silencing can inhibit cancer progression [21]. One other study has identified potential m^5^C sites in the mRNAs of human pancreatic cancer cells and observed NSUN2-mediated m^5^C mRNA metabolism in mouse models, suggesting a role in cancer progression and epithelial cell differentiation [22]. The report has also shown that NSUN2 promotes the proliferation of gastric cancer cells by inhibiting p57^Kip2^ in an m^5^C-dependent manner [23]. In addition, some RNA recognition proteins are involved in the regulatory effect of m^5^C modification on RNA, and these recognition proteins are called m^5^C-modified “readers”. For instance, Yang’s team discovered that NSUN2 and YBX1 drive the progression of bladder cancer by targeting m^5^C methylation sites in the untranslated regions of *HDGF*. YBX1 maintains the stability of its target mRNAs by recruiting ELAVL1 [18]. Additionally, studies have reported that YBX1 recognizes and binds to the m^5^C sites of *ORAI2* mRNA, enhancing its stability and promoting its expression. This, in turn, promotes the peritoneal metastasis and colonization of gastric cancer [24]. These studies demonstrate that YBX1 can serve as a “reader” of m^5^C, binding to mRNAs with m^5^C modifications, enhancing their stability, and subsequently elevating gene expression levels. Another m^5^C “reader,” ALYREF, can also promote the maintenance of various malignant phenotypes in tumors, including proliferation, metastasis, and drug resistance, through different regulatory mechanisms, such as pre-mRNA processing, mRNA stability, and nuclear-cytoplasmic shuttling. These studies demonstrate the crucial role of ALYREF in regulating gene expression through m^5^C modification [25–28]. Some research has identified the reasons for the elevated expression of NSUN2 in BC, which include decreased DNA methylation in the NSUN2 promoter region or alterations in gene copy number [29, 30]. Through database analysis, several studies have shown that NSUN2 is abnormally overexpressed in BC and indicates poor prognosis [31, 32]. Despite these findings, the precise regulatory mechanisms of NSUN2 and m^5^C modification in BC occurrence and progression remain unclear. This highlights the need for further investigation into the specific mechanisms underlying the impact of RNA m^5^C modification on BC pathogenesis. Consequently, our study focused on NSUN2 and employed RNA-BisSeq with a single-base resolution to identify *HGH1* as a potential m^5^C target in BC. We investigate the biological role of HGH1 in regulating BC progression and elucidate the mechanisms by which the key methylation molecule m^5^C regulates the expression of HGH1 through a series of experiments.

In this study, we initially demonstrate that NSUN2 significantly promotes the malignant phenotype of BC, which is partially dependent on its methyltransferase activity. Subsequently, through a combination of RNA-Seq and RNA-BisSeq, we identify *HGH1* as a gene that is highly expressed in BC and exhibits high levels of m^5^C. Both in vivo and in vitro experiments confirmed the oncogenic role of HGH1 in BC. Additionally, using MeRIP-qPCR and RIP-qPCR, we demonstrate the regulatory effect of the key m^5^C molecules NSUN2 and YBX1 on *HGH1*. Mechanistically, NSUN2 and YBX1 enhance the mRNA stability of *HGH1*, increasing overall protein synthesis efficiency, and synergistically regulating the expression of HGH1 in an m^5^C-dependent manner. Furthermore, we find that HGH1 may increase the overall translation efficiency of transcripts by binding to the translation elongation factor EEF2. Overall, this study reveals the mechanism of NSUN2 and its m^5^C-modified target HGH1, elucidating a regulatory pathway from post-transcriptional modification to protein translation, and suggesting that these proteins may serve as epigenetic markers and novel therapeutic targets for BC.

## Results

### Higher NSUN2 expression is significantly correlated with breast cancer and worse prognosis

**Fig. 1:**
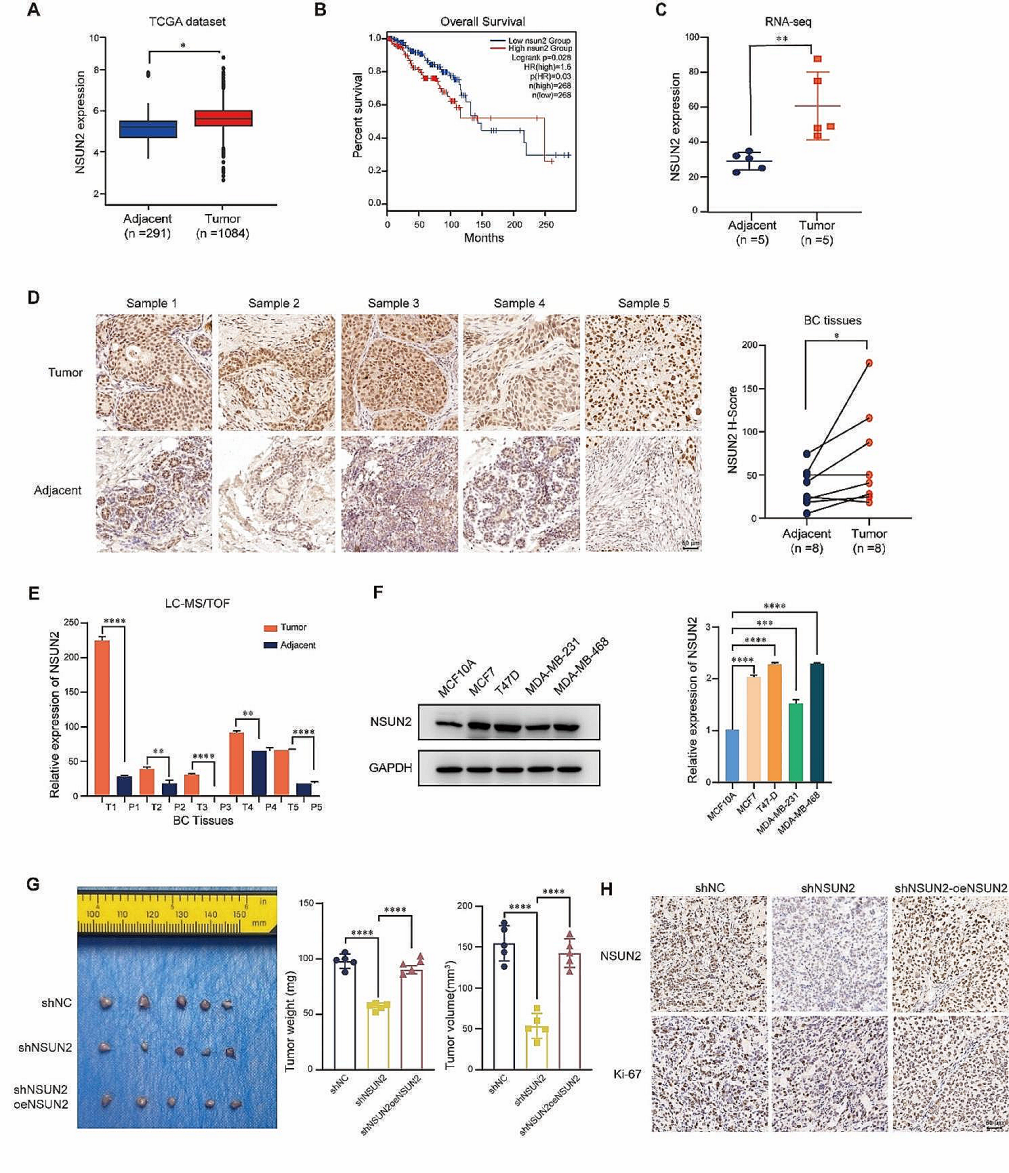
Higher NSUN2 expression in breast cancer samples. **(A)** NSUN2 expression in normal tissues and BC tissues from the TCGA dataset. **(B)** Plot of the overall survival probability of BC patients with high or low NSUN2 expression from the TCGA dataset. **(C)** NSUN2 mRNA expression in five clinical BC and adjacent tissue samples assessed by RNA-Seq (*n* = 5). **(D)** NSUN2 expression in five BC paraffin tissue samples. The samples were prepared and stained as described in the Materials and Methods (all images, 200×; *n* = 8). **(E)** LC-MS/TOF analysis of NSUN2 protein expression in five BC and adjacent tissue samples (*n* = 5). **(F)** Western blot analysis of NSUN2 expression in five breast cell lines. **(G)** The mammary fat pads of BALB/c nude mice (*n* = 5 per group) were surgically injected into the mammary fat pads with MCF7 cells, the tumors were weighed, and the tumor volume was measured. **(H)** Representative images of NSUN2 and Ki-67 expression in different groups of CDX tumors after IHC staining (all images, 200×). Data are mean ± SD (Standard deviation) of three independent experiments for (E) and (F). *p ≤ 0.05, **p ≤ 0.01, **p ≤ 0.001, ****p ≤ 0.0001 by t test of the indicated pairs or by log-rank (Mantel-Cox) test

To investigate the potential correlation between NSUN2 expression and the progression of BC, we first reviewed the TCGA (The Cancer Genome Atlas Program) datasets. As shown in Fig. 1A, NSUN2 was more highly expressed in tumor samples. Overall survival (OS) curves revealed that high expression of NSUN2 was associated with the development and poor prognosis of BC (Fig. 1B). RNA-Seq confirmed the increased protein levels of NSUN2 in five BC tissue samples compared with those in adjacent tissue samples. The same trend was observed by Immunohistochemistry (IHC) and liquid chromatography-mass spectrometry TOF (LC-MS/TOF). The results indicated that there was a significant difference in the expression level of NSUN2 between BC tissues and adjacent tissues (Fig. 1C and E, Fig. S1A-S1C). We also validated the higher expression of NSUN2 in 4 human BC cell lines (MCF7, T47D, MDA-MB231, and MDA-MB-468) than in a human mammary epithelial cell line (MCF10A) by Western blot (Fig. 1F). To determine whether NSUN2 facilitates BC progression, the MCF7-shNC, MCF7-shNSUN2, and MCF7-shNSUN2oeNSUN2 cell lines were used to establish the cell-derived xenograft (CDX) mice model, and tumor growth was assessed to estimate progression (Figs. S1D, E). As shown in Fig. 1G, the tumor volume was reduced in the shNSUN2 group and increased in the NSUN2 rescue group. In addition, the Ki-67 cell proliferation index was lower in the shNSUN2 group (Fig. 1H). These findings suggest that higher expression of NSUN2 indicates a poorer prognosis in BC.

### The m^5^C methyltransferase enzymatic activity of NSUN2 promotes breast cancer progression

**Fig. 2:**
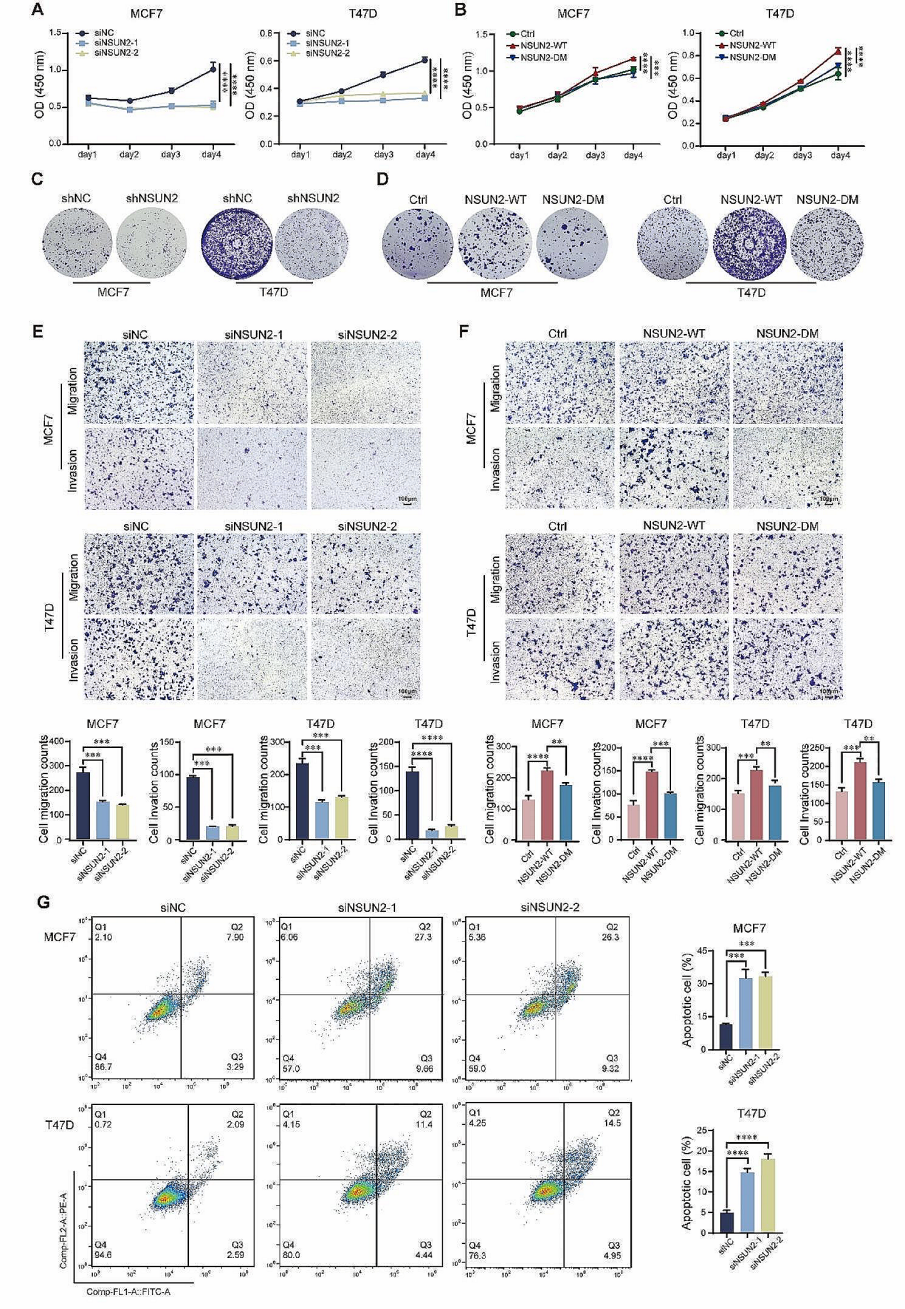
NSUN2-mediated promotion of breast cancer progression relies on methyltransferase enzymatic activity. **(A)** and **(B)** Cellular proliferation rates of BC cells with NSUN2 knockdown, NSUN2-WT or NSUN2-DM determined by CCK-8 assays and **(C)** and **(D)** colony formation assays. **(E)** and **(F)** Transwell assays were used to investigate the cell migration and invasion ability of NSUN2-knockdown, NSUN2-WT or NSUN2-DM cells. **(G)** FITC and PE fluorescence dyes were used to detect changes in cell apoptosis after NSUN2 knockdown by flow cytometry. Data are mean ± SD of three independent experiments for **(E-G)**. **p ≤ 0.01, ***p ≤ 0.001, ****p ≤ 0.0001 by t test

In addition to validating the ability of NSUN2 to promote the progression of BC in vivo, we also performed functional assays to investigate the phenotypes of BC cells regulated by NSUN2 in vitro. First, we constructed an NSUN2-overexpressing wild-type (NSUN2-WT) cell line and an NSUN2-double catalytic mutant (NSUN2-DM, C271A & C321A) cell line via a lentiviral transduction system to determine whether the effects of NSUN2 rely on its m^5^C methyltransferase enzyme activity (Fig. S1G). Cell counts kit-8 (CCK-8) assay was used to detect the cell growth curve. Figure 2A shows that cell growth was significantly suppressed after siRNA-mediated knockdown of NSUN2 (Fig. S1F). Moreover, overexpression of NSUN2 promoted cell proliferation (Fig. 2B). Similar results were obtained from the clonogenic assay, where NSUN2 knockdown significantly inhibited clonogenic activity, and this effect was reversed by overexpressing NSUN2-WT (Fig. 2C and D, Fig. S1H). We investigated the ability of NSUN2 to affect cell migration and invasion to evaluate the effect of NSUN2 on BC prognosis and tumor metastasis. The migration ability of the cells decreased after the knockdown of NSUN2. Consistently, the invasion of NSUN2 knockdown cells was also markedly inhibited, as determined by Transwell assays with Matrigel (Fig. 2E). The opposite results were observed in the overexpression group, especially in the NSUN2-WT group (Fig. 2F). Since the number of proliferating cells decreased upon NSUN2 knockdown, we further tested whether proliferation correlated with increased susceptibility to apoptosis. Flow cytometry analysis of the FITC and PE signals revealed that the number of apoptotic cells was increased in the siNSUN2-transfected group but not in the NC group (negative control siRNA) (Fig. 2G). Overall, these findings indicated that NSUN2 had a significant influence on BC cells, while the double catalytic mutant assay showed that the promotion effect on BC relied on the m^5^C methyltransferase activity of NSUN2.

### RNA-Seq combined with RNA-Bisseq screening identified *HGH1* as a potential target gene for m^5^C regulation in breast cancer

**Fig. 3:**
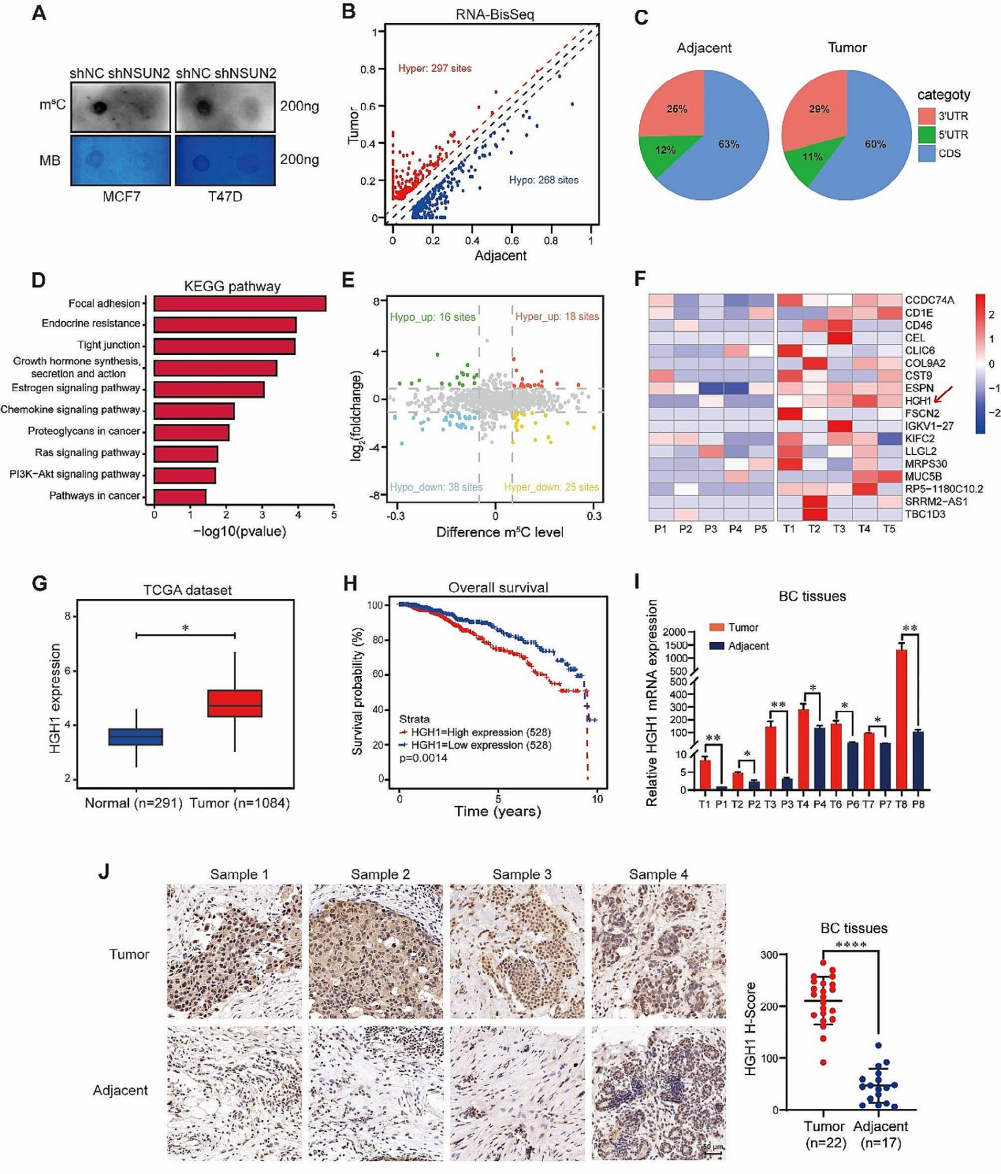
Screening of genes with abnormal m^5^C hypermethylation. **(A)** m^5^C dot blot assays using mRNA from MCF7 and T47D cells with or without NSUN2 knockdown. Methylene blue staining was used as a loading control. **(B)** Volcano plot showing differentially expressed m^5^C sites between tumor and adjacent tissues (*n* = 5). **(C)** Different proportions of m^5^C modifications in mRNA regions between BC tissues and adjacent tissues. **(D)** KEGG analysis revealed that m^5^C-hypermethylated genes with high expression levels in BC tissues were enriched in oncogenic signaling pathways. (**E)** Overlap of target genes from RNA-Seq and RNA-BisSeq of BC tissues (*n* = 5). The horizontal dashed line represents the threshold of the adjusted p-value of 0.05, while the vertical line represents the threshold of a log2-fold change greater than 1.5. **(F)** Clustering analysis heatmap showing the display level of different tissue samples. **(G)** Database analysis revealed an increase in HGH1 expression in tumor tissue. **(H)** Survival analysis showed that the group with high expression of HGH1 had a worse prognosis. **(I)** and **(J)** RT-qPCR and IHC experiments proved that HGH1 was expressed at higher levels in BC tissues than in paired adjacent tissues. Data are mean ± SD of three independent experiments for (I). *p ≤ 0.05, **p ≤ 0.01, ****p ≤ 0.0001 by t test or by log-rank (Mantel-Cox) test

As NSUN2 is a major methyltransferase responsible for RNA m^5^C modification, we explored the mechanism by which NSUN2 promotes the progression of BC. Then, we selected tissue samples from five clinical patients for transcriptome analysis combined with mRNA m^5^C bisulfite sequencing. First, we confirmed that NSUN2 can affect the overall methylation level of m^5^C in mRNA (Fig. 3A). Then, RNA-Seq combined with RNA-BisSeq was used to screen for mRNA targets modified by m^5^C in BC tissues. Bioinformatics analysis revealed 297 hypermethylation sites and 268 hypomethylation sites in BC tissues compared with adjacent tissues (Fig. 3B). Statistical analysis was conducted on the proportion of m^5^C modifications across various regions of the mRNAs. The coverage of m^5^C sites in the 5’-UTRs of both tumor tissues and adjacent tissues was similar. Notably, the 3’-UTR regions contained more m^5^C sites in the tumor tissues (Fig. 3C). Through Kyoto Encyclopedia of Genes and Genomes (KEGG) analysis of highly m^5^C-modified genes, several BC-related and tumor-related pathways, including the endocrine resistance, estrogen signaling, PI3K-Akt, and Ras signaling pathways, were found to be enriched in m^5^C modifications (Fig. 3D). Combined methylation and transcriptional data analysis revealed that in BC tissues, there were 18 sites with high methylation and high expression, 25 sites with high methylation and low expression, 16 sites with low methylation and high expression, and 38 sites with low methylation and low expression (Fig. 3E). We speculated that genes with high methylation and high expression in BC might have a stronger oncogenic effect. A cluster analysis heatmap revealed 18 genes with high methylation and high expression in BC (Fig. 3F). *HGH1* and *RP5-1180C10.2* exhibited high methylation levels in 80% of tumor tissues but low methylation levels in 80% of adjacent tissues. Since *RP5-1180C10.2* was a known lncRNA, we selected *HGH1* as a significant candidate gene among the 18 genes. Analysis of the TCGA database also revealed that HGH1 was highly expressed in breast tumor tissues (Fig. 3G). Moreover, patients with higher HGH1 expression had relatively worse survival than patients with lower HGH1 expression (Fig. 3H). We also collected clinical patient samples to validate that HGH1 was more highly expressed in BC tissues than in adjacent tissues at both RNA and protein levels (Fig. 3I and J).

### Suppression of HGH1 delays the progression of breast cancer

**Fig. 4:**
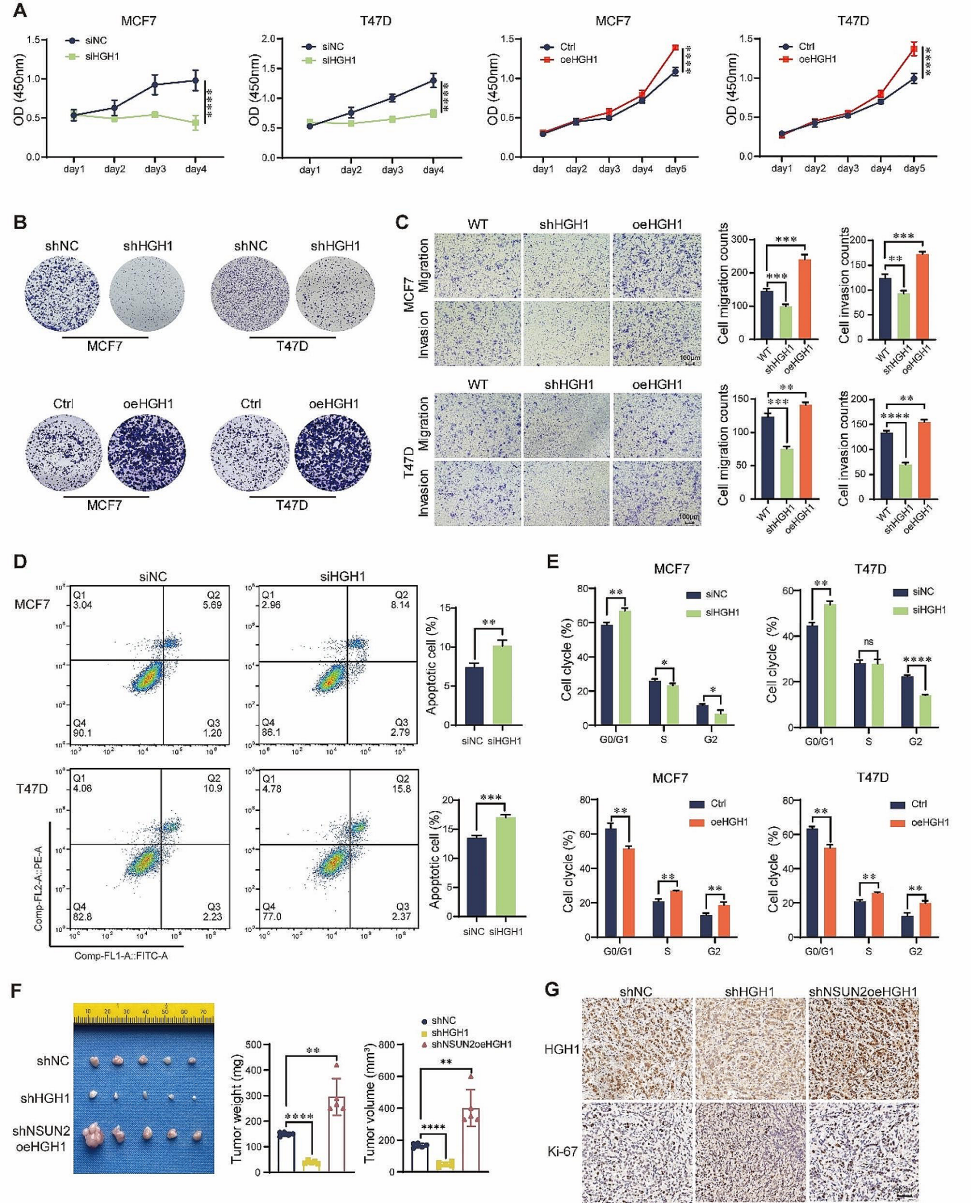
Suppression of HGH1 delays the progression of breast cancer. **(A)** CCK-8 assays were used to detect BC cell proliferation rates after interference with siHGH1 or oeHGH1. **(B)** Colony formation experiments detected the colony formation ability of BC cells after interference with shHGH1 or oeHGH1. **(C)** Transwell assays were used to assess the migration and invasion ability of BC cells after HGH1 knockdown. **(D)** FITC and PE fluorescence dyes were used to detect changes in cell apoptosis by flow cytometry after HGH1 knockdown. **(E)** PI fluorescence dye was used to detect cell cycle alterations by flow cytometry upon HGH1 knockdown or HGH1 overexpression. **(F)** The mammary fat pads of BALB/c nude mice (*n* = 5 per group) were surgically injected into the mammary fat pads with MCF7 cells, the tumors were weighed, and the tumor volume was measured. **(G)** Representative images of HGH1 and Ki-67 expression in different groups of CDX tumors after IHC staining (all images 200×) ns. no significant; Data are mean ± SD of three independent experiments for **(C-E)**. *p ≤ 0.05, **p ≤ 0.01, ***p ≤ 0.001, ****p ≤ 0.0001 by t test

Notably, HGH1 has not been previously reported to be associated with BC. To investigate whether HGH1 has a positive influence on BC progression, we constructed HGH1 knockdown cell lines and NSUN2 knockdown while rescuing HGH1expression cell lines (Fig. S2). Moreover, CDX models were used to test the function of HGH1 during BC carcinogenesis. CCK-8 assays revealed that BC cell proliferation decreased after HGH1 knockdown but increased after HGH1 overexpression (Fig. 4A). Similarly, the colony formation assay showed the same trend in BC cells after HGH1 interference (Fig. 4B). HGH1 also significantly improved the migration and invasion ability of BC cells (Fig. 4C). On the other hand, the number of apoptotic cells also significantly increased after HGH1 knockdown (Fig. 4D). As an important phenotype for characterizing cell life, we detected alterations in the cell cycle after HGH1 interference. With HGH1 knockdown, the proportion of BC cells in the G1 phase was significantly decreased, and the opposite trend was observed after HGH1 overexpression (Fig. 4E). The in vivo experimental results also showed that HGH1 promoted the progression of BC (Fig. 4F and G). Our experimental results preliminarily revealed the cancer-promoting effect of HGH1 in BC.

### HGH1 is a positive factor for translation efficiency in BC cells

**Fig. 5:**
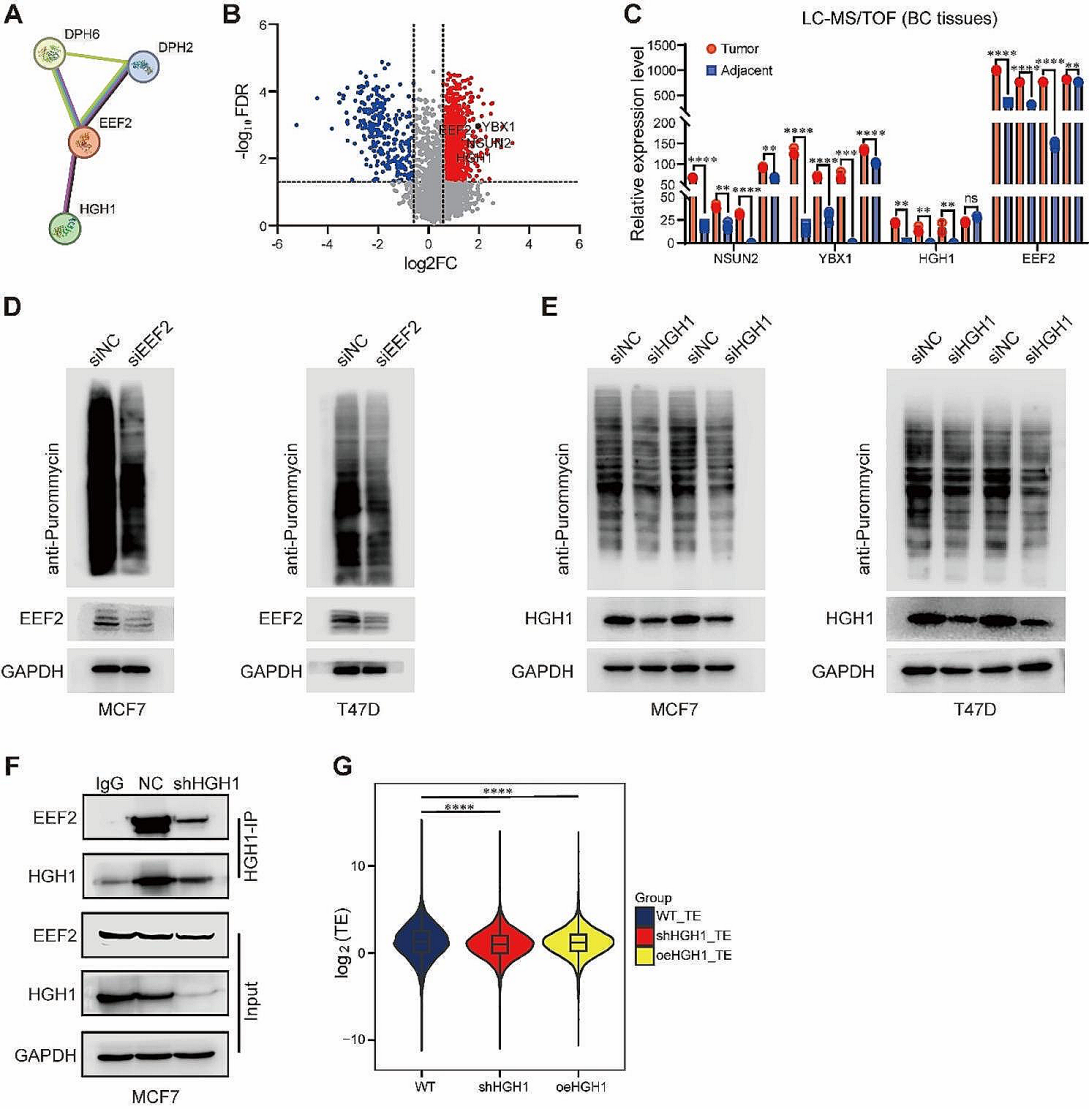
HGH1 affects translation efficiency in BC cells. **(A)** STRING database analysis showing a protein interaction relationship between EEF2 and HGH1. **(B)** and **(C)** Volcano plot and histogram of LC-MS/TOF data showing the high expression of EEF2 and HGH1 in BC tissues. **(D)** and **(E)** MCF7 and T47D cells transfected with siEEF2 (D) or siHGH1 (E) were treated with 1 µM puromycin for 1 h, and whole-cell lysates were subjected to western blot analysis with an anti-puromycin antibody. **(F)** Proteins were used in large excess to capture EEF2 from MCF7 cells by the anti-HGH1-protein A/G magnetic beads complex. The eluted material was analyzed by western blot and immunoblotting with anti-EEF2, anti-HGH1, and anti-GAPDH antibodies. **(G)** The average translation efficiency (TE) of MCF7 cells was analyzed via Ribo-Seq. ns. no significant; Data are mean ± SD of three independent experiments for (C) and (G). **p ≤ 0.01, ***p ≤ 0.001, ****p ≤ 0.0001 by t test

Currently, limited reports exist on the specific role of HGH1 in tumor progression. However, studies in yeast have revealed that Hgh1 can act as a molecular chaperone and bind to eukaryotic elongation factor 2 (eEF2) to form a complex involved in the process of translation elongation. The exact biological functions of HGH1 in human cancer are poorly understood and require further investigation. Hence, we explored the potential interaction between HGH1 and EEF2 using the STRING (https://cn.string-db.org/) database (Fig. 5A). Concurrently, proteomic mass spectrometry data were used to assess the expression profiles of HGH1 and EEF2 in BC tissues. Our findings revealed concurrent upregulation of both HGH1 and EEF2 in BC specimens (Fig. 5B and C). To further substantiate our observations, we conducted protein synthesis inhibition experiments and confirmed that EEF2 regulates overall protein translation efficiency in BC cells (Fig. 5D). Notably, a similar trend was detected in BC cells in which HGH1 expression was attenuated (Fig. 5E). To determine whether HGH1 can interact and form a complex with EEF2 in human BC cells, we performed co-immunoprecipitation (co-IP) experiments. As Fig. 5F clearly shows, there was a binding interaction between HGH1 and EEF2 in BC cells. Next, we further explored whether HGH1 can influence the overall translation efficiency of transcripts in BC cells. Ribo-Seq was employed to measure the quantity of mRNA that was being translated, while RNA-Seq served as a baseline for comparative calculations. It can be observed that the overall translation efficiency in BC cells decreased after HGH1 knockdown and increased after HGH1 overexpression (Fig. 5G). The findings presented above indicate that HGH1 plays a crucial biological role in regulating translation efficiency in human cancer cells, suggesting that it may be a potential mechanism contributing to BC progression.

### HGH1 is regulated by NSUN2 and dependent on its m^5^C methyltransferase activity

**Fig. 6:**
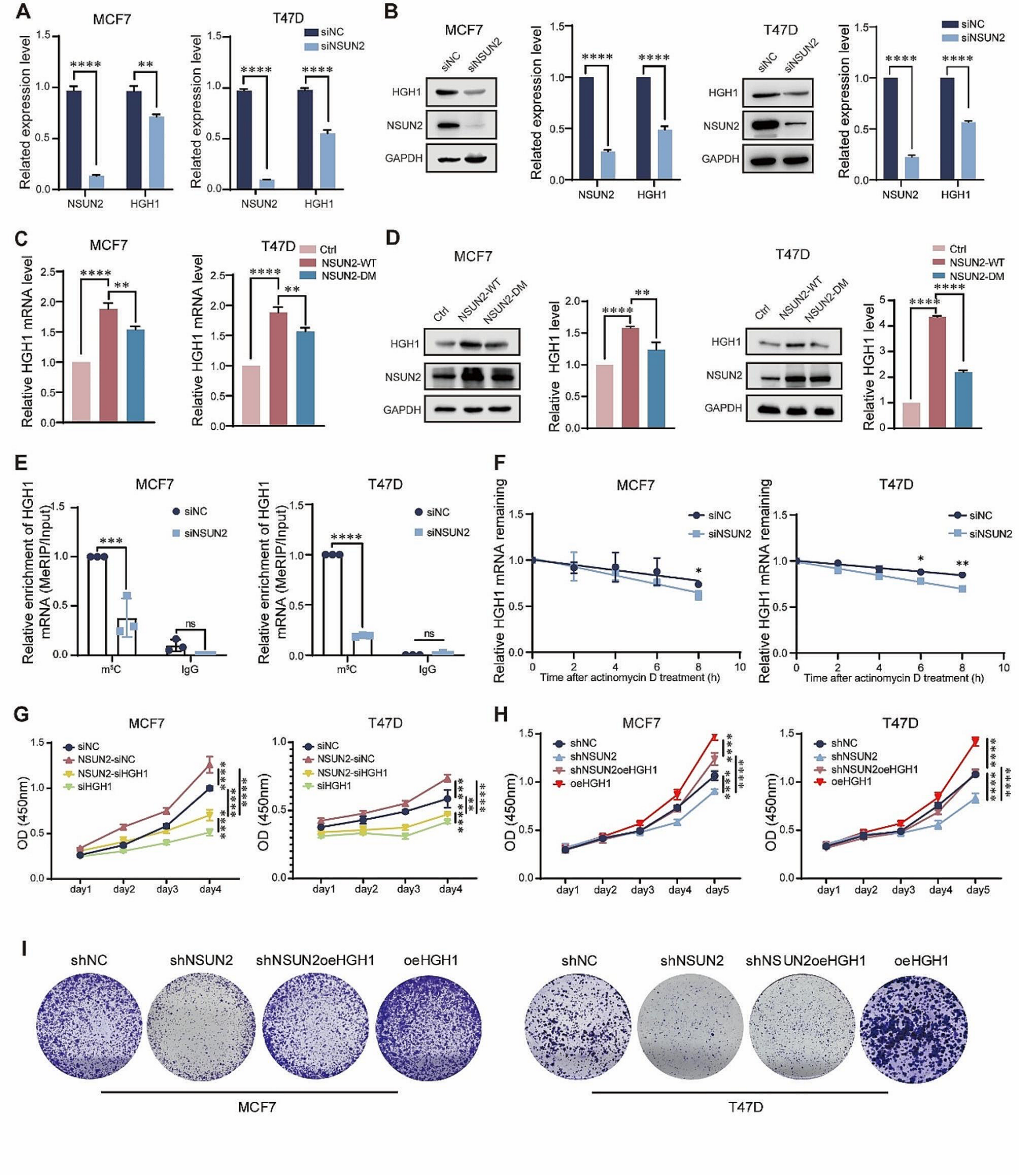
NSUN2-mediated mRNA m^5^C methylation promotes HGH1 expression **(A)** RT-qPCR assays were used to measure the expression of HGH1 in BC cell lines after NSUN2 knockdown, and **(B)** Western blot analysis was used to confirm the protein level. **(C) and (D)** RT-qPCR and Western blot assays were used to assess alterations in HGH1 expression after overexpression of NSUN2-WT or NSUN2-DM. **(E)** MeRIP-qPCR was used to measure the level of m^5^C on *HGH1* mRNA after NSUN2 knockout in BC cells. **(F)** Changes in the response to actinomycin D-mediated RNA synthesis inhibition and mRNA transcription interference were detected after NSUN2 knockdown. **(G)** and **(H)** CCK-8 experiments confirmed the influence of NSUN2 and HGH1 on the proliferation of BC cells and their downstream relationship. **(I)** A colony formation assay was used to determine the effect of restoring the expression of the downstream gene HGH1 in the NSUN2-knockdown cell line on the expansion ability of BC cells. ns. no significant; Data are mean ± SD of three independent experiments. *p ≤ 0.05, **p ≤ 0.01, ***p ≤ 0.001, ****p ≤ 0.0001 by t test

To determine the specific mechanisms underlying the association between HGH1 overexpression and the observed increase in m^5^C methylation, we investigated whether m^5^C methylation molecules participate in the regulation of this process. RT-qPCR and Western blot were used to assess alterations in HGH1 expression after NSUN2 interference. Figure 6A and Fig. S3A show the regulatory impact of NSUN2 on the gene expression of HGH1 following its knockdown. The regulatory role of the protein level is shown in Fig. 6B and Fig. S3B. Interestingly, both the RT-qPCR and Western blot results suggested that the NSUN2-mediated regulation of HGH1 expression was dependent on m^5^C methyltransferase activity (Fig. 6C and D). Then, we conducted a MeRIP-qPCR experiment using the m^5^C antibody, and as shown in Fig. 6E, the m^5^C on *HGH1* mRNA significantly decreased after NSUN2 knockdown. These results confirmed that NSUN2-mediated HGH1 regulation relies on m^5^C modification. Interestingly, we wondered why the decrease in mRNA m^5^C modification was correlated with a decrease in gene expression. Recent studies have shown that modifications to transcripts can affect their stability [33–36]. Specifically, certain RNA-binding proteins (RBPs) can recognize and bind to these modification sites, thereby affecting their translation. Therefore, we first validated that the degradation of HGH1 significantly increased after NSUN2 knockdown using an actinomycin D assay, thus preliminarily verifying that a reduction in m^5^C can reduce mRNA stability in BC cells (Fig. 6F). To explore whether NSUN2 can affect the progression of BC by regulating the expression of HGH1, we constructed a cell line in which NSUN2 was stably knocked down while HGH1 was overexpressed (Fig. S3C), and the NSUN2-overexpressing cell line in which HGH1 was knocked down. Figure 6G shows the effect of HGH1 on cell proliferation; that is, knocking down HGH1 in cell lines, even those overexpressing NSUN2, still weakened the proliferation ability of BC cells. As shown in Fig. 6H, HGH1 supplementation significantly restored the proliferation of BC cells in the NSUN2 knockdown cell lines; similar phenomena were also verified in the colony formation assay (Fig. 6I). These results confirm the upstream and downstream relationships between NSUN2 and HGH1 in the progression of BC. Because NSUN2 knockdown while overexpressing HGH1 in the rescue assay promoted tumor proliferation, it suggested to some extent that HGH1 may be a downstream gene of NSUN2.

### NSUN2 and YBX1 cooperate to regulate HGH1 expression by affecting mRNA stability and translation

NSUN2 can act as a methyltransferase to add m^5^C modifications to mRNA, and some RBPs can specifically recognize and bind to mRNA sites with methylations, as mentioned above. These RBPs are generally called “readers” in RNA methylation mode and play a role in regulating gene expression in different ways [37]. Because NSUN2 influences *HGH1* mRNA stability, we selected YBX1 as the main “reader” protein to explore its regulatory mechanism on HGH1 in BC and its synergistic effect on cancer promotion.

**Fig. 7:**
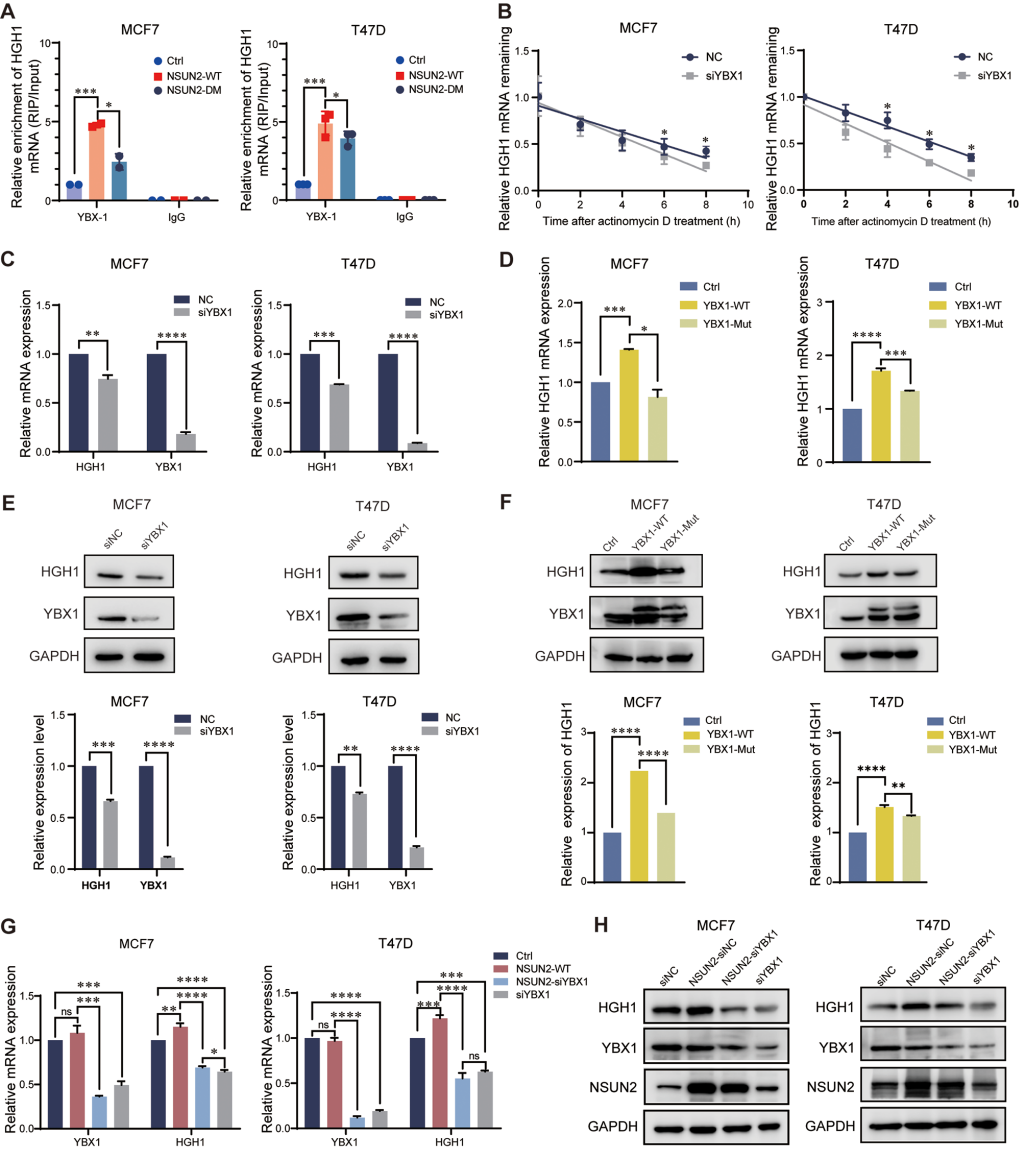
YBX1 is an important molecule of m^5^C regulating HGH1 expression in breast cancer. **(A)** YBX1 antibodies were used for RIP-qPCR to measure the level of YBX1 binding to HGH1 mRNA in the presence of NSUN2-WT. **(B)** Changes in the response to actinomycin D-mediated RNA synthesis inhibition and mRNA transcription interference after NSUN2 knockdown were detected. **(C)** RT-qPCR detection of the changes in the expression of the target gene HGH1 in the MCF7 and T47D cell lines after knockdown of siYBX1 and **(D)** overexpression of YBX1-WT and YBX1-Mut. **(E)** and **(F)** Western blot analysis of changes in protein levels. **(G)** and **(H)** RT-qPCR and Western blot experiments were performed to detect the synergistic effect of NSUN2 and YBX1 on the expression of the target gene HGH1. ns. no significant; Data are mean ± SD of three independent experiments. *p ≤ 0.05, **p ≤ 0.01, ***p ≤ 0.001, ****p ≤ 0.0001 by t test

**Fig. 8:**
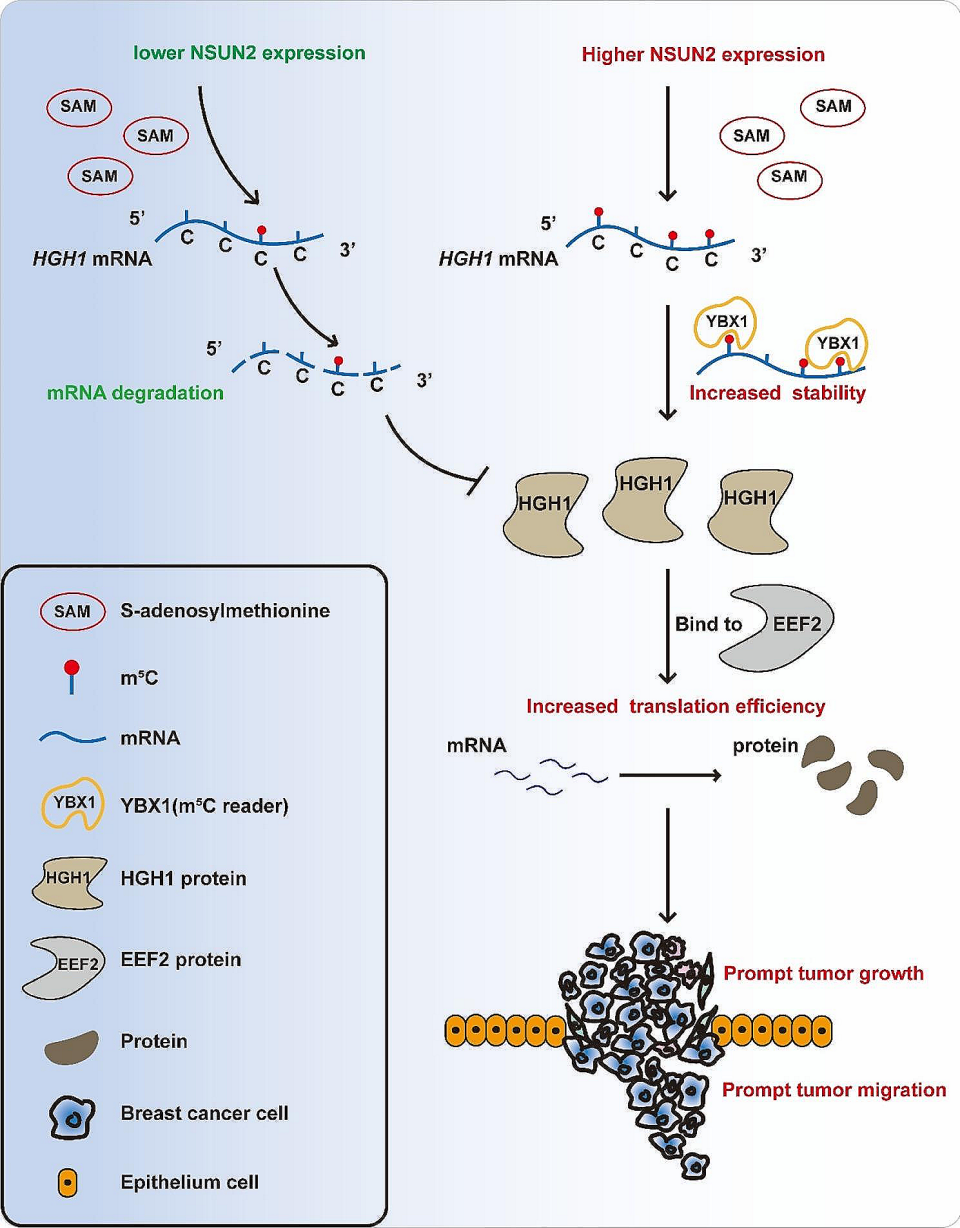
The mechanism diagram of this study

To investigate whether YBX1 can regulate the expression of HGH1 by recognizing m^5^C, we performed RIP-qPCR experiments and demonstrated that YBX1 not only binds to *HGH1* mRNA but also binds more strongly when NSUN2-WT is overexpressed (Fig. 7A). In addition, we also found that the mRNA stability of *HGH1* significantly decreased after knocking down YBX1 (Fig. 7B). Therefore, we constructed a YBX1 wild-type (YBX1-WT) stable overexpression cell line and an m^5^C binding site mutant (YBX1-Mut, W65A) cell line using lentiviral expression vectors. Knocking down YBX1 by siRNA significantly decreased the expression level of HGH1 (Fig. 7C), while overexpressing YBX1-WT significantly increased HGH1 expression (Fig. 7D). The same trend was also observed at the protein expression level (Fig. 7E and F). The above results indicated that the ability of YBX1 to regulate HGH1 expression depends to some extent on its function as a “reader” of RNA m^5^C modifications. According to mRNA stability experiments and existing research reports, methylations of mRNAs can affect translation and regulate protein expression. Therefore, we explored whether m^5^C modifications affect gene translation through protein synthesis experiments. We supplemented cultured cells with HPG (L-homopropargylglycine), with the amino acid being incorporated into proteins during active protein synthesis. Detection of the incorporated amino acid utilizes a chemoselective ligation or click reaction between an azide and alkyne, where the alkyne-modified protein is detected with Alexa Fluor 594. The results of the immunofluorescence experiment showed that after knockdown of NSUN2 (anti-NSUN2 with secondary in FITC fluorescent dye), protein synthesis in BC cells decreased (Alexa Fluor 594 fluorescent dye) (Fig. S4A). Puromycin incorporation experiments also confirmed that the synthesis of new proteins in BC cells was reduced after NSUN2 or YBX1 knockdown (Fig. S4B, C). The experimental results shown in Fig. 7G and H demonstrate the synergistic effect of NSUN2 and YBX1 on the regulation of HGH1 expression. We speculate that YBX1 can also contribute to BC progression. The results of the CCK-8 proliferation and cell apoptosis experiments revealed that YBX1 has a cancer-promoting effect on BC (Figure S5B, C). Flow cytometry experiments showed that YBX1 and HGH1 have the same trend of action in remodeling the cell cycle (Figure S5D). These findings open up new avenues for investigating the intricate interplay between m^5^C and tumor-related gene expression, which could have significant implications for our understanding of cancer biology (see Fig. 8).

## Discussion

In recent years, NSUN2 has been identified as being significantly overexpressed in various cancer types. Studies utilizing RNA-Seq, MeRIP-Seq, or Chip-Seq have revealed downstream target genes regulated by NSUN2 following interference in cell lines, elucidating the role of NSUN2 as a crucial regulator of RNA m^5^C modification that impacts disease progression by modulating gene expression [21, 23, 28, 38, 39]. Nonetheless, research on the mechanism of RNA m^5^C modification in BC is scarce. Notably, Zhidong Huang’s research demonstrated that high expression of NSUN2 in BC was correlated with poor prognosis based on TCGA database analysis [31]. Additionally, Ceshi Chen’s team revealed the involvement of the transcription factor KLF5 in regulating the progression of triple-negative breast cancer (TNBC). Through database analysis and in vitro and in vivo experiments, they verified that NSUN2/YBX1 enhances the stability of *KLF5* mRNA via m^5^C modification, leading to abnormal overexpression of KLF5 and exerting a pro-cancer effect [40]. In this study, we systematically investigated the oncogenic function of NSUN2 in BC, emphasizing its significance. By leveraging single-base resolution RNA-BisSeq and RNA-Seq, we conducted a comprehensive analysis of different subtypes of BC patient samples to identify *HGH1* as a target gene modified by m^5^C. Through MeRIP and RIP experiments, we demonstrated that NSUN2 and YBX1 enhance the mRNA stability of *HGH1* and cooperatively regulate its expression through m^5^C modification. Furthermore, we investigated the biological function of HGH1 in BC progression through proteomics analysis and translation sequencing experiments.

RNA m^5^C methylation is actively being studied and has been proven to regulate post-transcriptional modifications and affect protein translation, participating in many physiological and pathological processes. In eukaryotes, NSUN2 modifies m^5^C sites in tRNA’s variable arm and anticodon loop, stabilizing its secondary structure and affecting codon recognition [41]. Lack of m^5^C in tRNAs can cause fragmentation into 5’ tsRNAs, inhibiting protein translation [42]. Abnormal tsRNAs have been linked to breast and prostate cancer [43, 44]. In rRNA, m^5^C modification, primarily catalyzed by NSUN1 and NSUN5, affects ribosome biogenesis, polysome assembly and translation efficiency [45]. Whether m^5^C methylation on mRNA affects its translation process has been confirmed by numerous studies. A study reported that in cervical cancer, the mRNA of the anion channel protein LRRC8A, which is involved in cellular homeostasis, possesses m^5^C modifications and is regulated by the m^5^C “writer” NSUN2. m^5^C-modified *LRRC8A* mRNA binds to the RNA-binding protein YBX1, enhancing its stability and promoting its translation, thereby inhibiting cervical cancer cell apoptosis and promoting tumor development [38]. In our study, RIP-qPCR experiments confirmed that YBX1 indeed binds to the mRNA of *HGH1*, and this binding is increased in cells overexpressing wild-type NSUN2. This finding suggests specific recognition of the m^5^C modification on *HGH1* by YBX1, similar to the conclusions of many studies on YBX1 as an m^5^C “reader”. Therefore, this study focused on YBX1 as an m^5^C recognition protein and explored its potential regulatory role in BC. Our experimental results showed that even with the overexpression of NSUN2, the inhibition of YBX1 led to a significant reduction in the expression of HGH1. This indicates that NSUN2 alone is not sufficient for regulating HGH1 via m^5^C modification and that YBX1, an m^5^C reader, is crucial for the recognition and binding of *HGH1* mRNA to m^5^C. Although NSUN2 and YBX1 have been reported to act as oncogenes in some studies, their impact on the development of BC through m^5^C modification has not been reported. In this study, we confirmed that NSUN2/YBX1 can further enhance the malignant phenotype of BC, indicating that NSUN2 and YBX1 can exert carcinogenic effects by affecting mRNA stability consistent with existing research reports, and that the abnormal increase in m^5^C in tumors is also similar to the findings of other cancers. Moreover, we preliminarily clarified that NSUN2 may regulate the key target gene *HGH1* through m^5^C modification, thereby affecting the progression of BC.

HGH1 is a highly conserved armadillo repeat protein, and its role in human physiology and pathology has rarely been reported. To date, only one study has shown that high expression of HGH1 can promote the proliferation, migration, and epithelial-mesenchymal transition of colorectal cancer, but the biological function of HGH1 in human tumor cells remains unclear [46]. In this study, we first verified the high expression of HGH1 in BC patients and its association with reduced survival. Further in vitro and in vivo experiments demonstrated that knocking down HGH1 could inhibit the malignant progression of BC. Experiments involving the overexpression of NSUN2 with simultaneous knockdown of HGH1, as well as the stable knockdown of NSUN2 with HGH1 replenishment, demonstrated the upstream and downstream relationships between NSUN2 and HGH1 in regulating BC progression, highlighting the significant impact of HGH1. Additionally, we noted two studies reporting the biological function of Hgh1 in yeast. A study by Leonie Moünkemeyer revealed that Hgh1, as a molecular chaperone, can recruit TRic (chaperonin) during translation elongation to collaboratively promote the folding of eEF2 in the ribosomal translation process, acting as a multidomain protein dependent on chaperone proteins [47]. Research by Florian H. Schopf revealed that in yeast, Hgh1 can form a complex with Hsp90 and Cns1 as molecular chaperones to jointly promote ribosomal translation folding of eEF2 [48]. eEF2 is an essential elongation factor in the ribosomal translation process of eukaryotes that interacts with ribosomes to stabilize intermediate states [49, 50]. Our proteomic analysis and bioinformatics analysis of BC revealed that HGH1 and EEF2 are co-expressed at a higher level in tumor tissue than in adjacent tissue and that their expression levels are correlated. Preliminary protein synthesis inhibition experiments showed that knocking down EEF2 or HGH1 significantly reduced overall protein synthesis in BC cells, and co-IP confirmed the binding of HGH1 to EEF2 in BC cells. We speculate that HGH1 may play a similar biological role in human breast cells as in yeast cells, that is, by participating in the formation of translation elongation complexes to affect the translation of proteins. Therefore, we used cell lines with silenced or overexpressed HGH1 to perform a combined Ribo-Seq and RNA-Seq analysis to determine whether HGH1 can affect the translation of human BC cells. The results showed that silencing HGH1 significantly reduced overall translation efficiency in BC cells, while overexpressing HGH1 significantly increased translation efficiency. These results validate our hypothesis and preliminarily clarify the biological role that HGH1 may play in the progression of BC.

The key innovations and findings of this study are twofold. First, we verified that the promotion of BC progression by NSUN2 is partially dependent on its m^5^C methyltransferase activity. By collecting BC tissues from patients and combining RNA-Seq and RNA-BisSeq data, we selected m^5^C modification sites and identified the key target *HGH1* at the single-base resolution level. Second, through in vivo and in vitro experiments, we elucidated the molecular mechanism by which NSUN2/YBX1 depends on m^5^C to regulate the stability and expression of *HGH1* mRNA. Additionally, we not only demonstrated the promoting effect of HGH1 on the progression of BC but also preliminarily explored the biological function of HGH1 in human BC cells through co-IP and Ribo-Seq. However, breast cancer is highly heterogeneous [51–53], and we also found differences in the expression of NSUN2 in different subtypes of BC tissues in our study. In the future, whether NSUN2 and m^5^C modification targets can serve as biomarkers for subtypes of BC requires further study. We will conduct in-depth studies at the molecular structural level of nucleic acids and proteins to explore the underlying mechanisms involved in the process from post-transcriptional modification to protein translation. Additionally, we aim to include a larger number of clinical patients, collect samples including fresh tumor tissue, blood, and urine, and perform multiangle joint analysis to verify whether RNA m^5^C modification can serve as a diagnostic marker for tumors and an epigenetic therapeutic target.

In summary, this is the first study to elucidate the molecular mechanism of RNA m^5^C modification mediated by NSUN2 in BC by regulating the downstream target HGH1, revealing a regulatory pathway from post-transcriptional modification to protein translation, suggesting that NSUN2 and HGH1 may become new epigenetic markers and potential therapeutic targets for BC​​.

## Materials and methods

### Bioinformatics analysis

The TCGA database (https://www.cancer.gov/ccg/research/genome-sequencing/tcga*)* was used to analyze the differences in NSUN2 and HGH1 expression between normal and tumor tissues of the breast. Survival plots related to NSUN2 and HGH1 expression in BC patients were generated with Gepia2 (http://gepia2.cancer-pku.cn/). The STRING database (https://cn.string-db.org/*)* was used to analyze proteins that interact with HGH1.

### Clinical samples

Thirty-nine BC tissues and nine adjacent tissues were collected to detect the NSUN2 protein level by IHC. These samples were preserved by pathologists via paraffin embedding. Five pairs of BC and adjacent tissue samples were collected for enrichment of mRNA and library construction, which were sufficient for RNA-Seq and RNA-BisSeq. These samples were stored in liquid nitrogen until use. All the clinical tissue samples were collected from 2019 to 2020 at the First Affiliated Hospital of Zhengzhou University. This study was approved by the Institutional Review Board of the First Affiliated Hospital of Zhengzhou University (the approval number 2019-KY-31) and complied with the Declaration of Helsinki.

### Immunohistochemistry

First, the paraffin samples were heated at 60 °C for 1 h. Then, an automatic immunohistochemistry instrument (ROCHE) and an automatic staining instrument (LEICA) were used to incubate the sections with the antibodies. Images were scanned using the 3DHISTECH PANNORAMIC VIEWER. Concentrations of anti-NSUN2 (Sigma, HPA037896), anti-HGH1 (Proteintech, ab181031), and anti-Ki67 (Servicebio, GB111499) were used according to the supplier’s instructions.

### LC-MS/TOF

RIPA lysis buffer was used to extract total protein from the breast tissues, and a 10 K ultrafiltration tube was used for FASP digestion. Mass spectrometry analysis was carried out using Eksigent NanoLC 400 liquid chromatography and AB Sciex6600*TripleTOF mass spectrometry. The human protein database was downloaded from the UniProt website, SWATH mass spectrometry data were imported into DIA-NN 1.8.1 software for database comparison analysis, and the corresponding protein data of the peptides were obtained for further analysis. Based on three biological replicates of each sample, the differentially expressed proteins with a fold change > 1.5 or < 0.67 and a p-value < 0.05 were screened for further research.

### Cell lines

The human normal breast epithelial cell line MCF10A and the human breast cancer cell lines MDA-MB-468, MDA-MB-231, and MCF7 were purchased from the National Collection of Authenticated Cell Cultures of the Chinese Academy of Sciences. The human breast cancer cell line T47D was kindly provided by Dr. Yue Du’s laboratory in the Department of Pharmacy, First Affiliated Hospital of Zhengzhou University.

### Animals

According to the guidelines of the Hospital Ethics Committee, this project purchased 6-week-old female BALB/c nude mice from Jiangsu JICUI Yiming Biotechnology Co., Ltd. The mice were raised in an SPF-grade environment at the Experimental Animal Center in Henan Province, with an environmental temperature of 21–23 °C and a humidity of 40-60%.

### CDX mode

Six-week-old female BALB/c nude mice were used for xenotransplantation experiments. The experimental nude mice were anesthetized via isoflurane inhalation (1%) according to the animal ethics requirements. After complete anesthesia, the constructed cell lines were injected into the fat pad of the second mammary gland on the right side of the nude mice. After the injection, the wound was sutured, and the nude mice were observed until they woke up and regained their ability to move. The mice were observed every 3 days, and the nude mice were sacrificed based on the size of the tumor. The tumor size and weight were recorded, and the tumors were fixed in a 4% paraformaldehyde tissue cell fixation solution. After paraffin embedding and sectioning, immunohistochemical detection was performed.

### Plasmids and transfection

The PLKO.1-shNSUN2 plasmid was kindly provided by Prof. Yun-Gui Yang (Beijing Institute of Genomics, Beijing, China). The 3FLAG-EGFP-NSUN2-WT, 3FLAG-EGFP-NSUN2-DM (C271/321A), 3FLAG-EGFP-YBX1-WT, 3FLAG-EGFP-YBX1-Mut (W65A), LV-HGH1-RNAi, and LV-HGH1 lentivirus were purchased from GENECHEM (Shanghai, China). Transient transduction was carried out using Lipofectamine 3000 (Invitrogen) according to the manufacturer’s instructions. The cells were then cultured for 48 h and harvested for western blot analysis. For lentiviral transduction, a second-generation lentivirus packaging system consisting of psPAX2 (Addgene) and PMD2. G (Addgene) was used to generate virus particles. In brief, plasmids were transfected into HEK293T packaging cells at 60% confluence using Lipofectamine 3000 (Invitrogen) according to the manufacturer’s instructions. After an additional 48 h of incubation, the supernatant was collected, filtered through a 0.45-µm filter (Millipore), and used to infect host cells in the presence of 6 µg/mL HitransG A/P (GENECHEM). The resulting stable polyclonal populations of transduced cells were then selected with puromycin or hygromycin (Solarbio, China) for two weeks, followed by validation by western blotting.

The shRNA sequences used are as follows:

shNSUN2#1: 5’- GCTGGCACAGGAGGGAATATA-3’;

### RNA interference

Cells were seeded at a confluence of 40% in 6-well or 96-well plates. After 24 h, according to the manufacturer’s instructions, the cells were transfected with the indicated siRNA oligonucleotides using Lipofectamine RNA iMAX (Invitrogen). Then, the cells were cultured for 72 h and harvested for cell viability assays or Western blot analysis. The target sequences of the siRNA oligonucleotides used are as follows:

siNSUN2#1: 5’- GAGAUCCUCUUCUAUGAUCTT-3’;

siNSUN2#2: 5’- CACGUGUUCACUAAACCCUAUTT-3’;

siYBX1#1: 5’- GGAUAUGGUUUCAUCAACATT-3’;

siHGH1: 5’- AUGCUUGUUGAAGCCAUCATT-3’;

siEEF2: 5’-GCCCUCUUAUGAUGUAUAUTTAUAUACAUCAUAAGAGGGCTT-3’.

### RNA extraction and real-time RT-PCR (RT-qPCR)

Total RNA was extracted from cells and tissues using TRIzol reagent (Invitrogen). Then, the RNA was reverse-transcribed into complementary DNA (cDNA) using a PrimeScript RT reagent kit (Takara, RR037A). RT-qPCR analysis was conducted using iTaq Univer SYBR Green Supermix (Bio-Rad) and QuantStudio5 Applied Biosystems (Thermo Fisher Scientific) according to the manufacturer’s instructions. The results were analyzed as the fold change in expression. The primer pairs used are listed below:

NSUN2 forward: 5’- CAAGCTGTTCGAGCACTACTAC-3’,

NSUN2 reverse: 5’- CTCCCTGAGAGCGTCCATGA-3’;

YBX1 forward: 5’- GCGGGGACAAGAAGGTCATC-3’;

YBX1 reverse: 5’- CGAAGGTACTTCCTGGGGTTA-3’;

HGH1 forward: 5’- ACATCCGCAAGATGCTTGTTG-3’;

HGH1 reverse: 5’- GAAGGATCAGGTAGGCTCCCT-3’;

GAPDH forward: 5’- GGAGCGAGATCCCTCCAAAAT-3’;

GAPDH reverse: 5’- GGCTGTTGTCATACTTCTCATGG-3’;

18S rRNA forward: 5’- GTAACCCGTTGAACCCCATT-3’;

18S rRNA reverse: 5’- CCATCCAATCGGTAGTAGCG-3’.

### RNA m^5^C dotblot

Total RNA was extracted from cells using the TRIzol method, and mRNA was enriched using the magnetic bead method. After quantification with Qubits, 200 ng of mRNA from each group of cells was divided 3–5 times and spotted onto a Hybond-N + nylon membrane (Solarbio, Cat# YA1760). Each spot was crosslinked with UV for 5 minutes in a dark crosslinking instrument. After all the samples were crosslinked, the membrane was blocked with 5% nonfat milk and then incubated with an anti-m^5^C antibody (1:1000, Abcam, ab214727) overnight at 4 °C. The next day, the membrane was incubated with a secondary antibody at room temperature, and the membrane was exposed to the imaging system using a sensitive ECL exposure solution (Fisher Biotech). After exposure, the membrane was stained with 0.02% methylene blue (Sangon Biotech) to determine whether the amount of sample loaded was consistent for each group.

### RNA-seq and RNA-Bisseq

Total RNA was extracted using the TRIzol method. The mRNA was enriched using the VAHTS mRNA Capture Beads Kit (Vazyme, 401-01/02) according to the manufacturer’s instructions. DNase I (NEB, M0303) and RNaseOut (Invitrogen, 10,777,019) were used to remove DNA and RNase from the enriched mRNA. RNA fragmentation reagents (Ambion, AM8740) were used to fragment the mRNA. Magnetic RNA Clean Beads (Vazyme, N412) were used to purify the mRNA samples, and Qubits was used to determine the mRNA concentration.

A KAPA stranded mRNA-seq kit (KK8429) was used to construct mRNA sequencing libraries according to the manufacturer’s instructions. The main steps included cDNA synthesis of the first and second strands, purification of the second strand synthesis products, A-tailing addition, adapter addition, purification of the ligation products, cDNA library amplification, and purification of the PCR products of the library.

mRNA C-T base conversion was performed according to the instructions of the conversion reagent (Zymo, R5001), unmethylated C was converted to U, and C was converted to T after PCR, while methylated C remained unchanged; 1 µL of diluted Luciferase Control RNA (Promega, L456A) was added as an internal reference for C-T conversion to evaluate conversion efficiency. The obtained mRNA was mixed with random primers (ACT hexamers), and cDNA I strand synthesis was performed using 100 mM DTT, 2.5 mM dNTPs, and SuperScript II. For chain II synthesis and subsequent steps, refer to the transcriptome process.

The libraries were transported on dry ice and sent to the Nanjing Jiangbei New Area Biomedical Public Service Platform for Illumina NovaSeq 6000 platform-based transcriptome sequencing.

#### Sequencing data analysis

The adaptors and low-quality bases were trimmed using Cutadapt and Trimmomatic software, respectively. Clean reads were mapped to the human genome (hg19) using meRanTK40’s meRanGh. The Dhfr spike-in was used to calculate the C to T conversion rate of > 99%. For each sample’s BAM file, only high-quality bases (Q ≥ 30) were used for variant calling. The mismatched types at each site were inferred based on the gene annotations on the chain. Additionally, each variant had to meet the following criteria: base quality score ≥ 30, mismatch frequency ≥ 0.1, and C + T coverage ≥ 20. Furthermore, we required that (i) the variant still met the above criteria, (ii) the variant’s signal ratio was ≥ 0.9, and (iii) the p-value was calculated using a one-sided binomial test. To identify high-confidence sites, we required that each tissue type site pass the p-value < 0.001 according to Stouffer’s Z score method. The annotation of m^5^C sites was performed using BEDTools intersectBed. The m^5^C sites that satisfied the conditions in each sample were merged, and consecutive C sites were removed. The mean methylation level of each tumor methylation site was calculated.

Combining the transcriptome sequencing data, the genes were divided into four groups: high methylation and high expression, high methylation and low expression, low methylation and high expression, and low methylation and low expression, based on the criteria of |diffmean (Tumor-Normal)| ≥ 0.05, |log2foldchange| ≥ 1, MeanT_P > 0.1, and Bs_seq_p-value < 0.05. Differentially significant genes with high methylation and high expression were selected as potential target genes for BC m^5^C dependency.

### MeRIP-qPCR

Then, 5 µg of m^5^C antibody was mixed with 50 µl of Protein A Dynebeads in 200 µl of IPP Buffer and incubated at room temperature for 1 h. Total RNA was extracted from the samples using the TRIzol method, and mRNA was enriched using magnetic beads. DNA and RNA enzymes were removed from the enriched mRNAs using DNase I (NEB, M0303) and RNaseOut (Invitrogen, 10,777,019). The mRNA was fragmented using RNA fragmentation reagents (Ambion, AM8740). The mRNA samples were purified using RNA Clean Beads magnetic beads (Vazyme, N412). The concentration of mRNA was measured using Qubits, and 1/10 of the mRNA was used as the input control. The mRNA was mixed with magnetic bead antibodies and incubated at 4 °C for 4 h on a rotator. The supernatant was discarded, and the magnetic beads were washed three times with IPP buffer. Then, 200 µl of PK buffer and 40 µl of protease K were added, mixed well, and incubated at 55 °C with shaking at 1100 rpm for 60 min. After centrifugation, the supernatant was discarded, and the supernatant was transferred to a new 1.5 ml centrifuge tube. Then, 240 µl of RNA extraction reagent (ACMEC, AC13320) was added, mixed well, and incubated at 30 °C with shaking at 1100 rpm for 5 minutes. The upper layer was aspirated to a new 1.5 ml centrifuge tube. One microliter of glycogen, 20 µl of sodium acetate, and 660 µl of absolute ethanol were added. The samples were stored overnight at -80 °C. After centrifugation at 4 °C and 13,300 rpm for 30 min, the supernatant was carefully discarded, and the blue precipitate was RNA. The RNA was dissolved in an appropriate amount of nuclease-free water. For each input sample and MeRIP sample, the same amount of mRNA was reverse transcribed. The reverse transcription reaction mixture was prepared according to the instructions of the Novogene low-starting amount reverse transcription kit (HiScrip III All-in-one RT SuperMix Perfect for qPCR; R333), and RT-qPCR was subsequently performed.

#### RNA stability experiment (actinomycin D treatment method)

Cells were plated in 6-well plates and treated with actinomycin D (5 µg/mL, MedChemExpress) for various durations: 0, 2, 4, 6, and 8 h. Equal numbers of BC cells were collected at each time point, and total RNA was extracted using TRIzol reagent (Invitrogen). RNA from each sample was reverse-transcribed into cDNA, and RT-qPCR was used to measure the mRNA level of HGH1. The relative abundance of mRNA at each time point was calculated in reference to the t = 0 time point.

#### RIP-qPCR

The cell pellet was immersed in RIP lysis buffer (composed of 150 mM KCl, 10 mM HEPES (pH 7.6), 2 mM EDTA, 0.5% NP-40, 0.5 mM DTT, protease inhibitor, and RNase inhibitor) on ice for 30 min. One-tenth of the cell lysate was designated as the input control. The remaining cell lysate was then incubated either with rabbit IgG-coated beads or with anti-YBX1 (Abcam, ab76149)-coated beads for 4 h at room temperature. After this incubation, the bead-antibody-protein-RNA complex was washed five times with ice-cold washing buffer (consisting of 200 mM NaCl, 50 mM HEPES (pH 7.6), 2 mM EDTA, 0.05% NP-40, 0.5 mM DTT, and RNase inhibitor). Subsequently, the immunoprecipitated sample was digested with proteinase K, and the RNA was precipitated using glycogen (Thermo Scientific, AM9516). Total RNA was then extracted using TRIzol reagent, followed by RT-qPCR analysis.

#### Protein synthesis assay (Click-iT HPG Alexa Fluor method and anti-puromycin method)

Protein synthesis was detected using the Click-iT HPG Alexa Fluor 594 Protein Synthesis Kit (Thermo Fisher, C10429) according to manufacturer’s instructions. Briefly, the cells were treated with Click-iT HPG Alexa Fluor working solution and subjected to a single wash following formalin fixation. Then, cells underwent a 20-minute permeabilization period with 0.5% Triton X-100, and the cells were washed twice with 3% BSA in PBS. Next, a reaction cocktail was added to each well, followed by a 30-minute incubation at room temperature, shielded from light, and the cells were then washed with Click-iT reaction rinse buffer. After a 30-minute blocking period with 5% BSA, the cells were incubated with an anti-NSUN2 primary antibody followed by a FITC fluorescent secondary antibody. Finally, after washing with PBS, the cells were stained with Hoechst for 30 min. The samples were then examined using a fluorescence microscope equipped with appropriate filters for DAPI/Hoechst, FITC, and Alexa Fluor 594. Another method was applied in vitro using puromycin (P8230, Solarbio, China). Cells were treated with 1 µM puromycin for 1 h. Then, the cells were collected, and the proteins were extracted. Protein expression was determined through Western blotting.

### Co-IP (co-Immunoprecipitation)

The protein of BC cells was extracted. NP-40 and Triton X-100 were used to prepare the lysis solution, and protein quantification was performed after 10 min of ice lysis. One hundred micrograms of protein from each group of samples was added to 500 µl of lysis solution. Part of it is used as input, while the other part is used as IP and IgG samples. Then, 20 µl of magnetic beads were added to each sample, 500 µl of TBS and 5 µg of the corresponding antibodies (HGH1 as the IP group and IgG as the negative control group) were added, and the mixture was incubated at room temperature for 1 h. After incubation, 100 µg of protein sample was added to the antibody magnetic bead suspension and incubated overnight with 4° rotation. The magnetic scaffold was used to discard the supernatant, and the protein lysis solution was used to lyse the magnetic bead-antibody protein mixture. We determined whether there was an interaction between the two proteins through Western blot.

### Ribo-Seq

Two hundred microliters of ribosomal buffer (20 mM Tris-Cl, 150 mM NaCl, 5 mM MgCl2, 1 mM DTT) and 10% Triton X-100, 100 mM DTT, 50 mg/ml cyclohexide, 1 U) were added to 2 × 10^7^ cells, and the resulting solution was combined with the µ-cracking solution prepared with L DNase. After gently blowing and beating, the ice was shaken for 10 min, and the mixture was regularly reversed. The samples were centrifuged at 4 ℃ and 20,000 × g for 10 min, after which the supernatant was transferred to a new centrifuge tube precooled on ice. Then, 120 µl of lysate was digested at room temperature with 3 µl of RNase I (Life Technologies, AM2294) for 45 min to remove RNA that was not protected by ribosomes. Finally, digestion was terminated with 4 µl of Superase In (Life Technologies, AM2694). RNA was extracted from the sample using TRIzol LS (Life Technologies, 10296-010), and residual ribosomal RNA was removed from the sample using a Ribo-Off rRNA Removal Kit (Vazyme, N406-01). Then, 16% urea PAGE was used to separate the sample RNA, the target RNA band position was 27–30 nt, and a Small RNATM PAGE Recovery Kit (Zymo R1070) was used for gel cutting and recovery. Finally, the Small RNA Amplification Library Prep Kit for Illumina (Vazyme, NR801) was used for library construction.

### Electronic supplementary material

Below is the link to the electronic supplementary material.

Supplementary Material 1

Supplementary Material 2

Supplementary Material 3

Supplementary Material 4

Supplementary Material 5

Supplementary Material 6

Supplementary Material 7

## Data Availability

No datasets were generated or analysed during the current study.

## Abbreviations

m^5^C: 5-methylcytosine
BC: Breast cancer
TCGA: The Cancer Genome Atlas
NSUN: NOP2/Sun domain
YBX1: Y -box binding protein 1
IHC: Immunohistochemistry
CDX: Cell-derived xenograft
RT-qPCR: Real-time RT-PCR
MeRIP: Methylated RNA immunoprecipitation
RIP: RNA immunoprecipitation
RNA-seq: RNA sequencing
RNA-BisSeq: RNA bisulfite sequencing
Ribo-Seq: Ribosome sequencing
EEF2: Eukaryotic elongation factor-2
CNA: Copy Number Alterations
tsRNA: trans-splicing RNA
