# Supplementary figures and images for: NSUN2/YBX1 promotes the progression of breast cancer by enhancing HGH1 mRNA stability through m5C methylation
Source: Breast Cancer Res. 2024 Jun 6;26:94. doi: 10.1186/s13058-024-01847-0 (PMC11155144; doi:10.1186/s13058-024-01847-0)

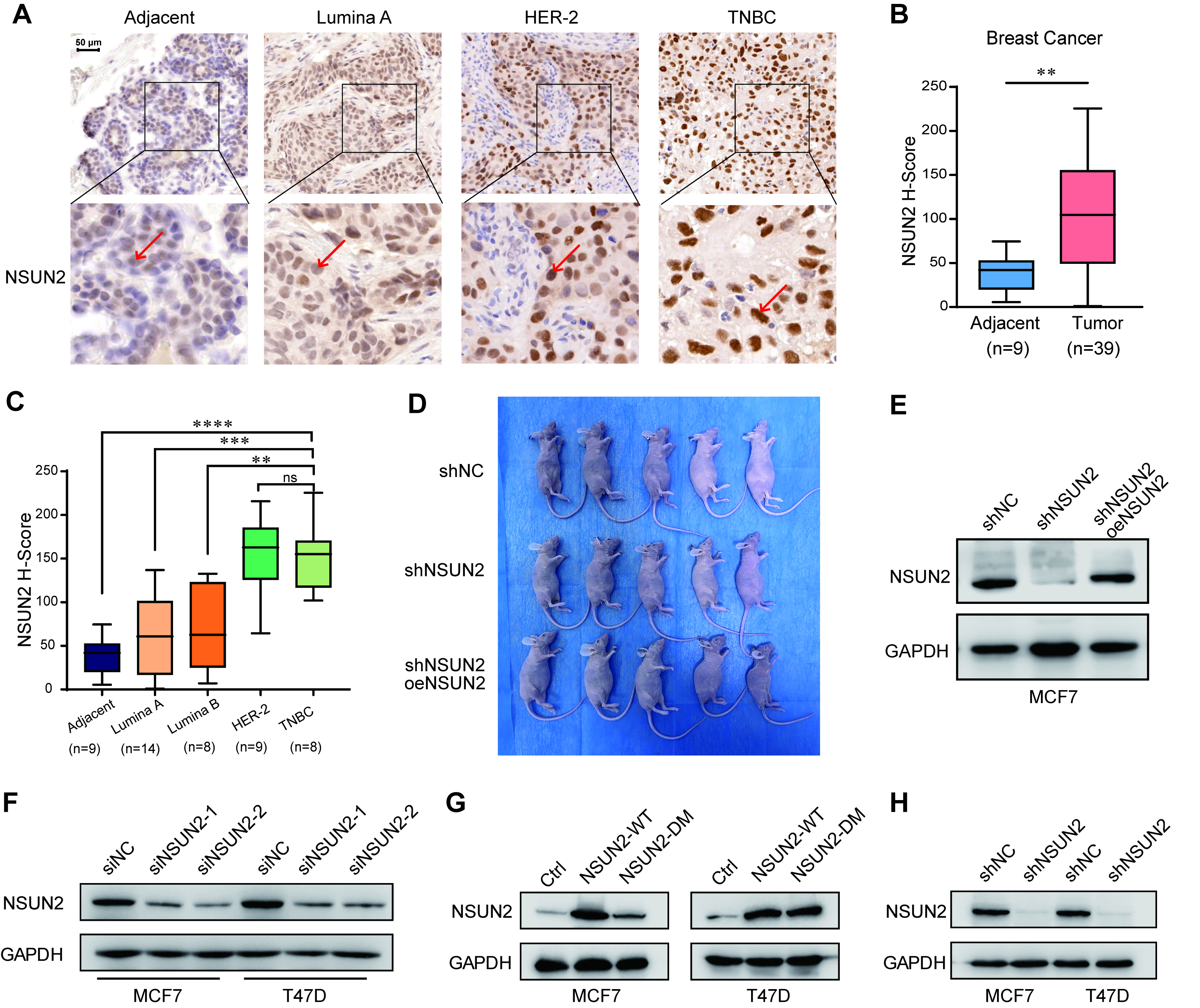

Supplement: Supplementary file 1 — Supplementary Material 1 [file 13058_2024_1847_MOESM1_ESM.tif]

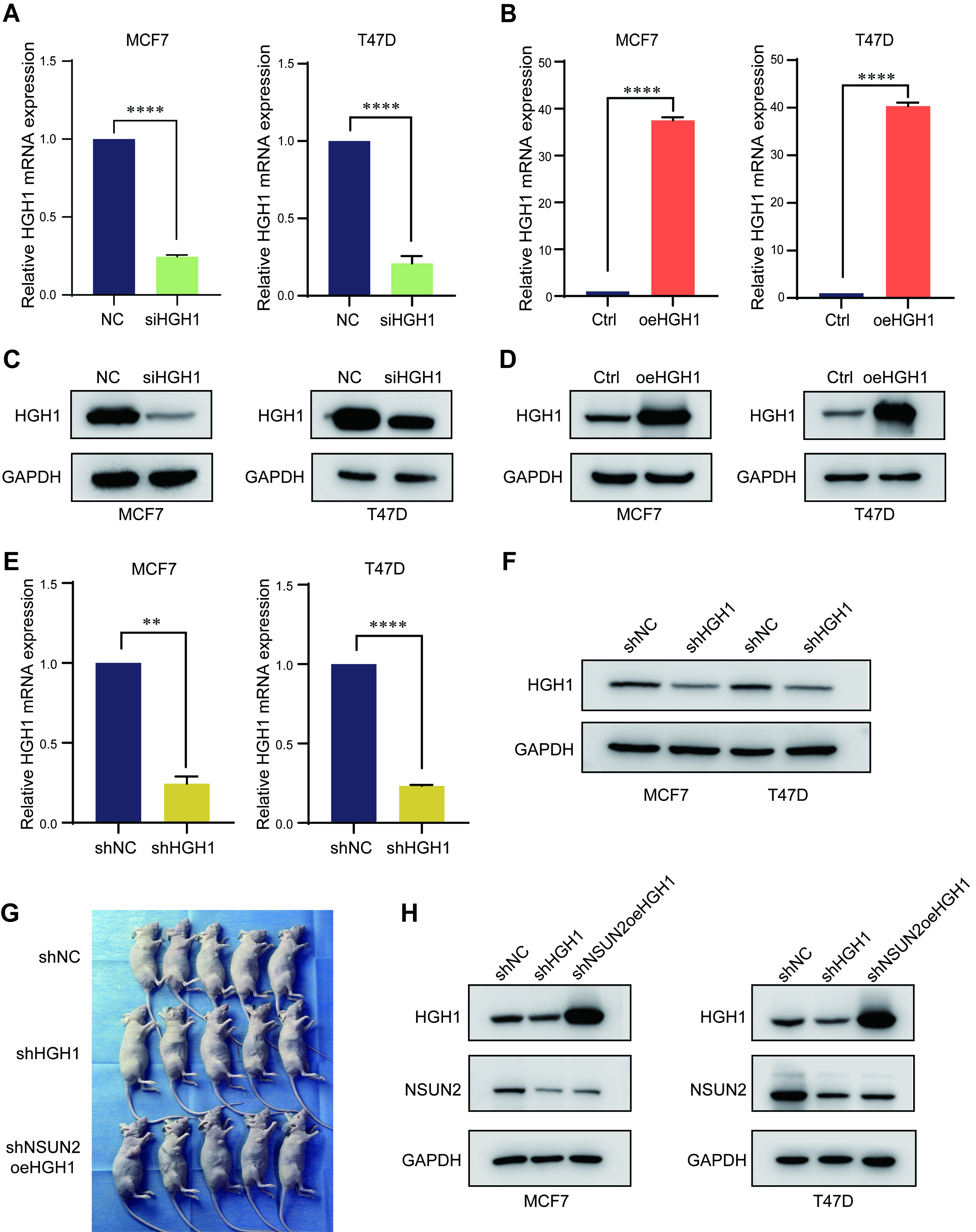

Supplement: Supplementary file 2 — Supplementary Material 2 [file 13058_2024_1847_MOESM2_ESM.tif]

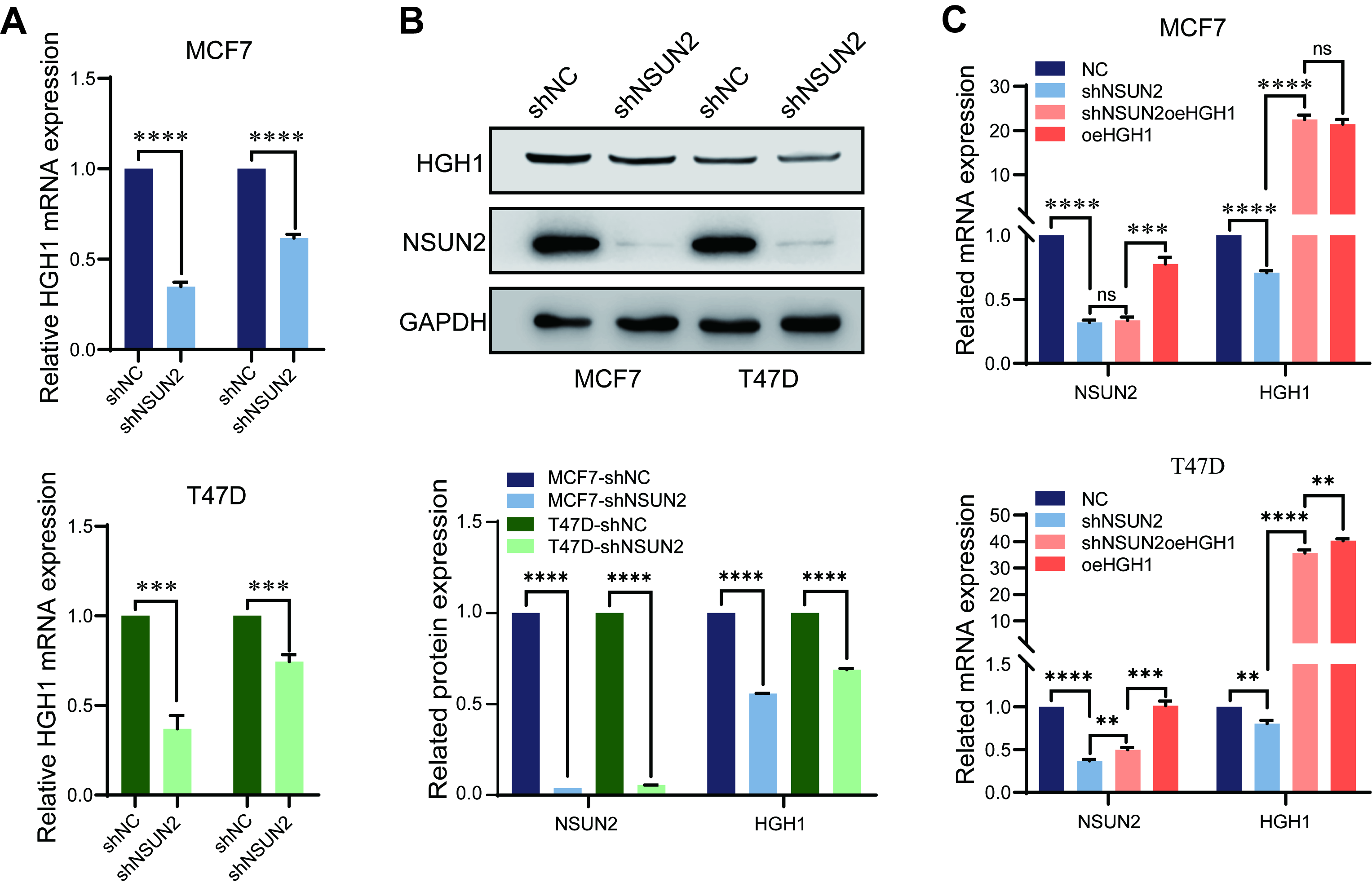

Supplement: Supplementary file 3 — Supplementary Material 3 [file 13058_2024_1847_MOESM3_ESM.tif]

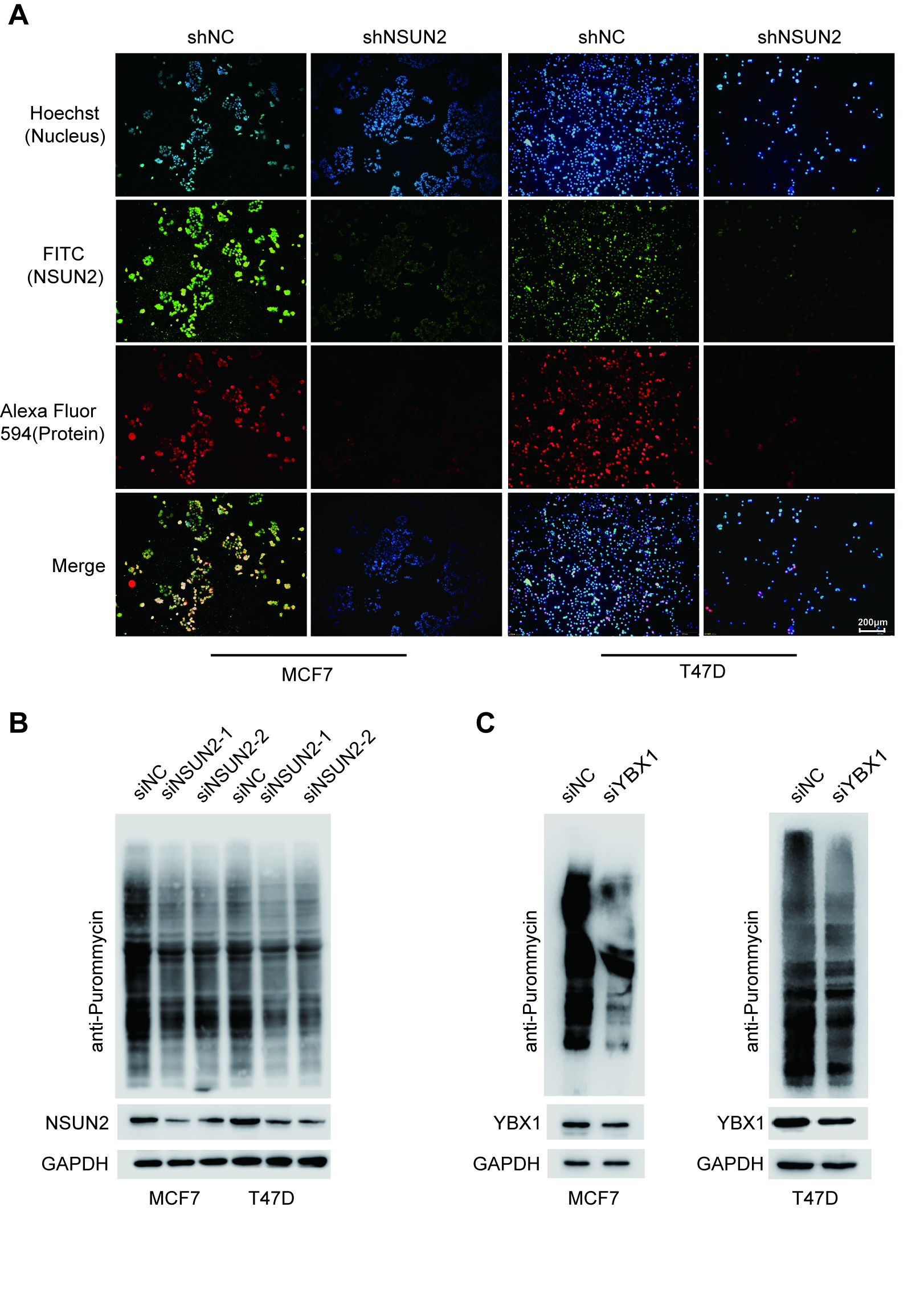

Supplement: Supplementary file 4 — Supplementary Material 4 [file 13058_2024_1847_MOESM4_ESM.tif]

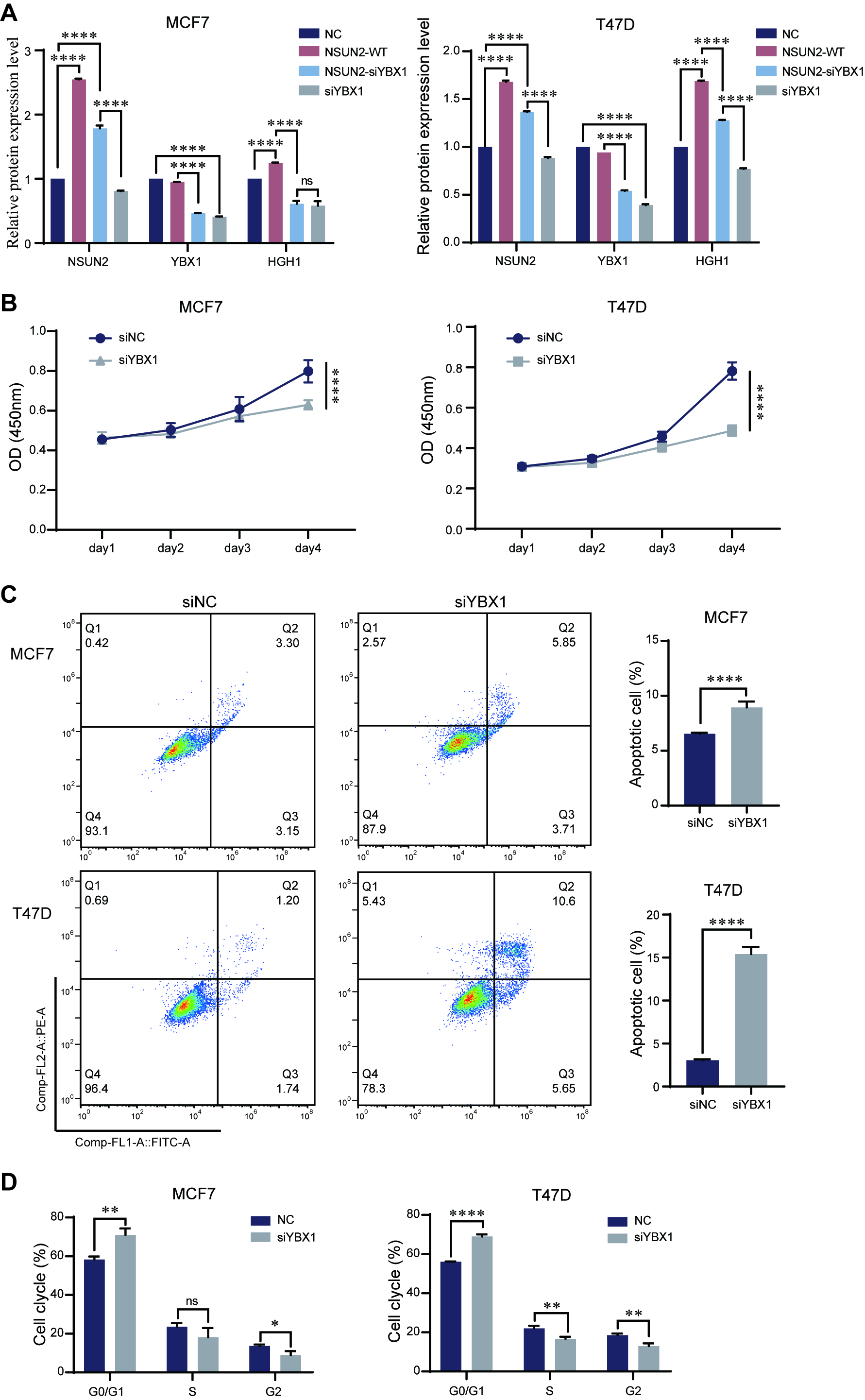

Supplement: Supplementary file 6 — Supplementary Material 6 [file 13058_2024_1847_MOESM6_ESM.tif]
