# Supplementary material for: NSUN2/YBX1 promotes the progression of breast cancer by enhancing HGH1 mRNA stability through m5C methylation
Source: Breast Cancer Res. 2024 Jun 6;26:94. doi: 10.1186/s13058-024-01847-0 (PMC11155144; doi:10.1186/s13058-024-01847-0)
Supplement: Supplementary file 5 — Supplementary Material 5 [file 13058_2024_1847_MOESM5_ESM.docx]

**Fig. 1F**

|  | **lane 1-lane 10:**  **MCF10A, MCF7, T47D, MDA-MB-231, MDA-MB-468; MCF10A, MCF7, T47D, MDA-MB-231, MDA-MB-468** |
| --- | --- |
| **GAPDH**  **（37 kd）** | 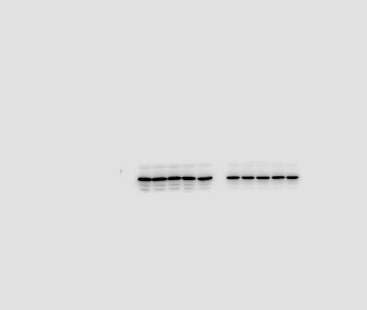 |
| **NSUN2**  **（110 kd）** | 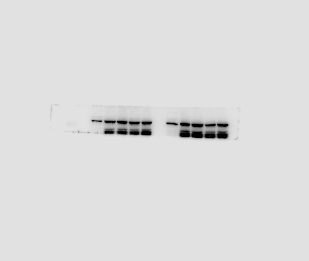 |

**Fig. 3A**

|  | **samples: MCF7shNC, MCF7shNSUN2** | **samples: T47DshNC, T47DshNSUN2** |
| --- | --- | --- |
| **Anti-m^5^C** | 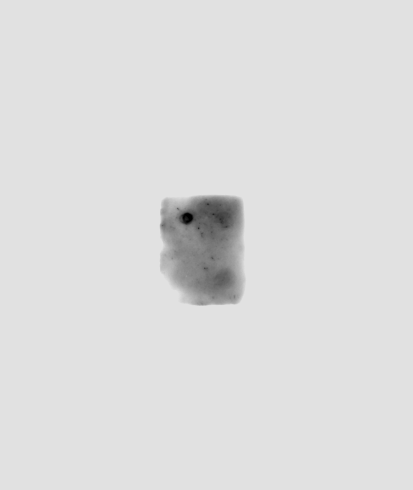 | 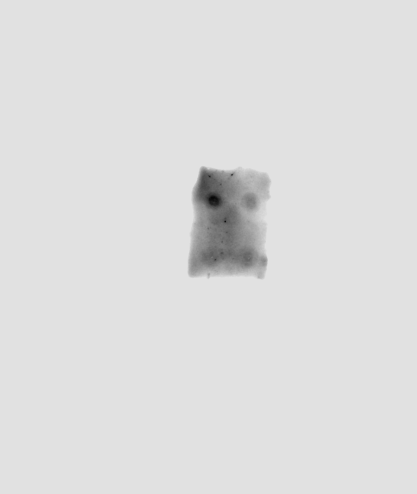 |
| **Methylene blue** | 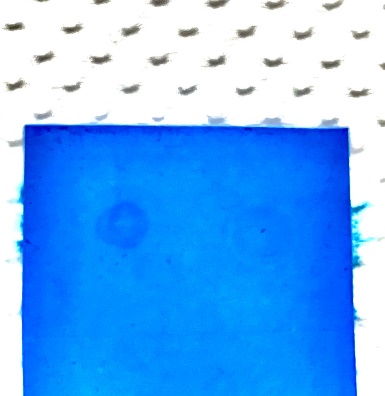 | 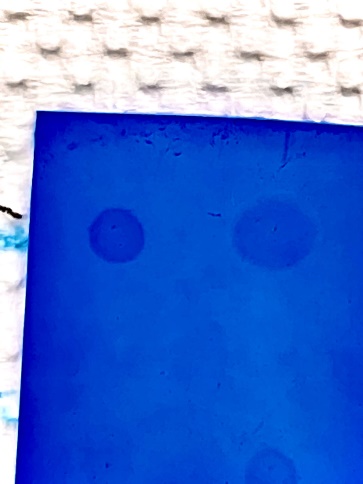 |

**Fig. 5D**

|  | **lane 1-lane 2: MCF7-siNC, MCF7-siEEF2** | **lane 1-lane 2: T47D-siNC, T47D-siEEF2** |
| --- | --- | --- |
| **Anti-puro** | 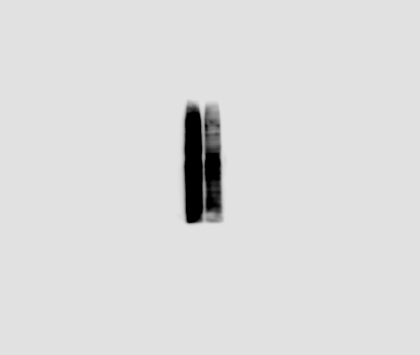 | 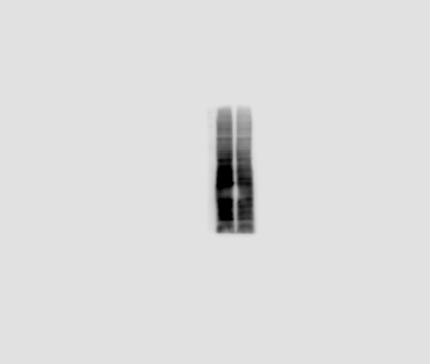 |
| **GAPDH**  **（37 kd）** | 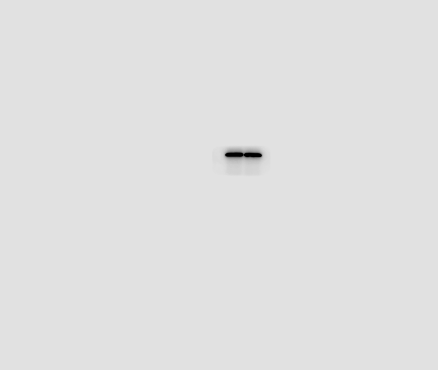 | 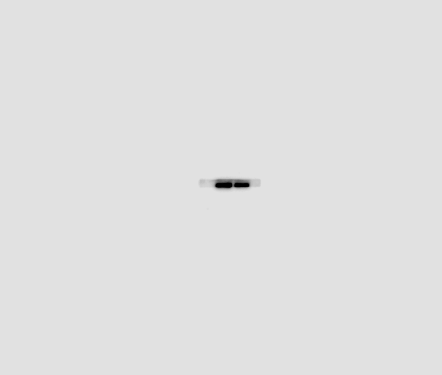 |
| **EEF2**  **(95 kd)** | 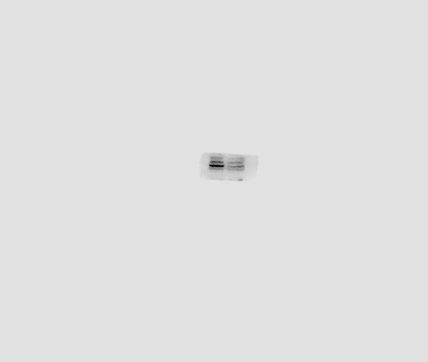 | 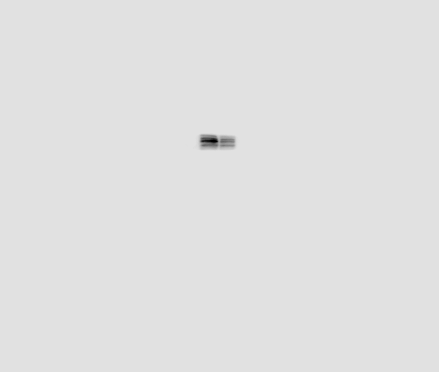 |

**Fig. 5E**

|  | **lane 1-lane 8: MCF7-siNC, MCF7-siHGH1; MCF7-siNC, MCF7-siHGH1; MCF7-siNC, MCF7-siHGH1; MCF7-siNC, MCF7-siHGH1;** | **lane 1-lane 4:**  **T47D-siNC, T47D-siHGH1; T47D-siNC, T47D-siHGH1;** |
| --- | --- | --- |
| **Anti-puro** | 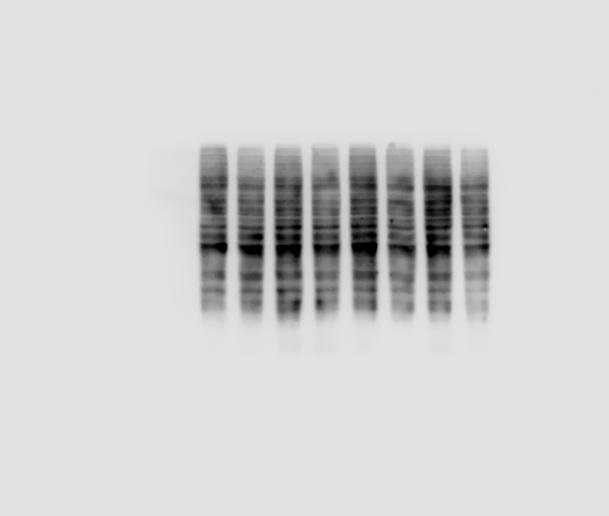 | 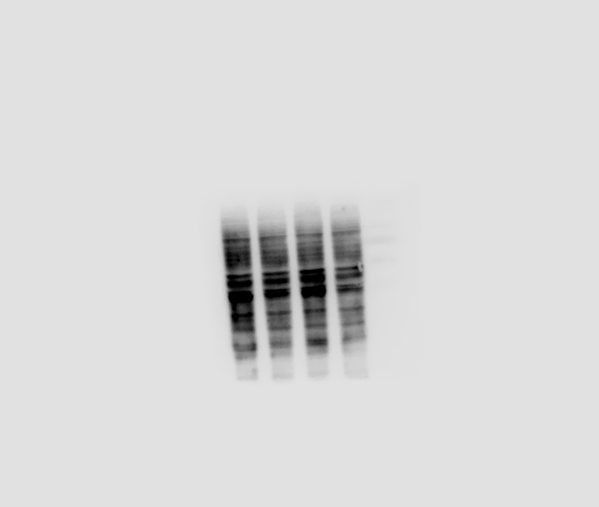 |
| **GAPDH**  **（37 kd）** | 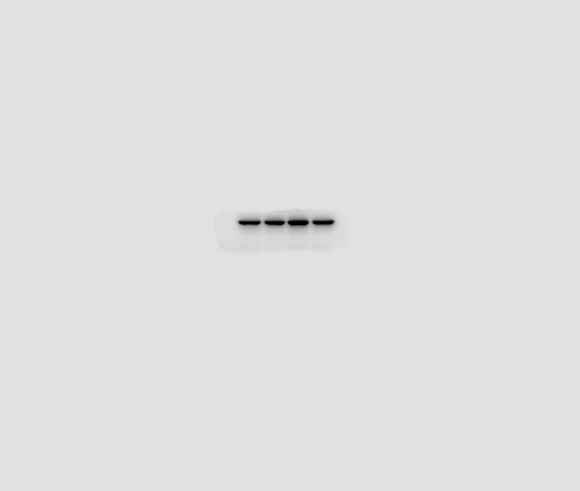  **(Due to insufficient sample size, we only repeated GA twice with MCF7-siNC and MCF7-siHGH1)** | 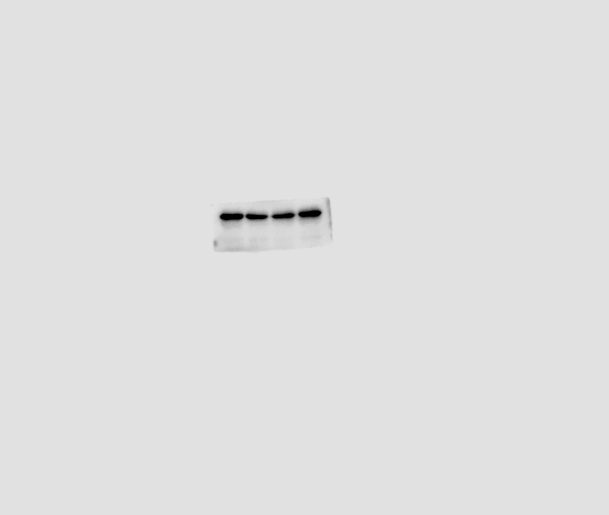 |
| **HGH1**  **(50 kd)** | 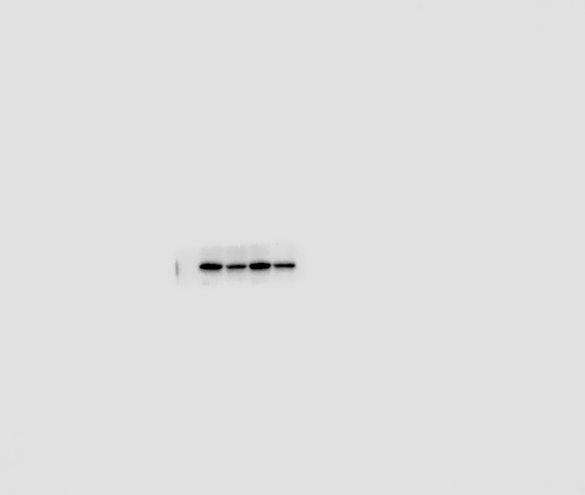  **(Due to insufficient sample size, we only repeated HGH1 twice with MCF7-siNC and MCF7-siHGH1)** | 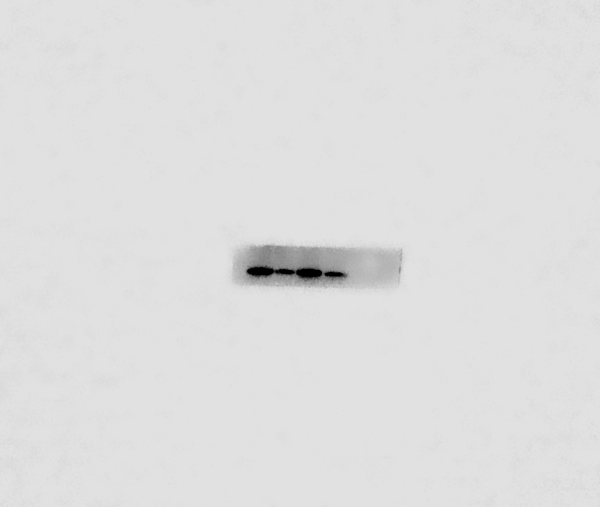 |

**Fig. 5F**

|  | **lane 1-lane 3:**  **IgG-IP, MCF7shNC, MCF7shHGH1** |
| --- | --- |
| **EEF2 (HGH1-IP)**  **（95 kd）** | 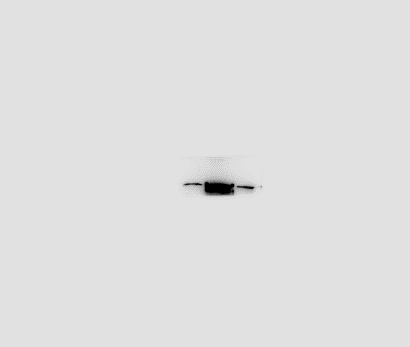 |
| **HGH1(HGH1-IP)**  **（50 kd）** | 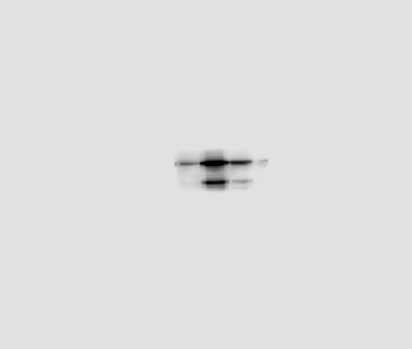 |
| **EEF2 (Input)**  **（95 kd）** | 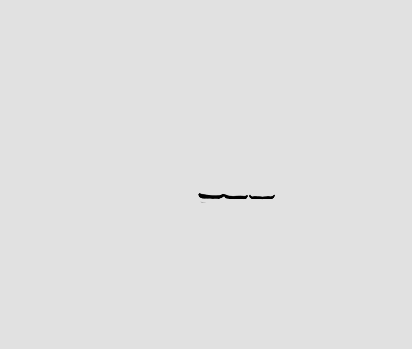 |
| **HGH1 (Input)**  **（50kd）** | 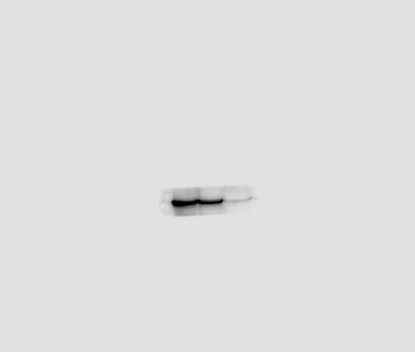 |
| **GAPDH (Input)**  **（37 kd）** | 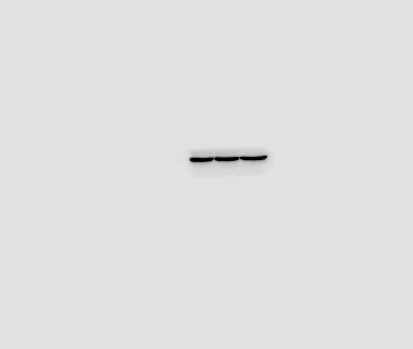 |

**Fig. 6B**

|  | **lane 1-lane 2: MCF7-siNC, MCF7-siNSUN2** | **lane 1-lane 2: T47D-siNC, T47D-siNSUN2** |
| --- | --- | --- |
| **HGH1**  **(50 kd)** | 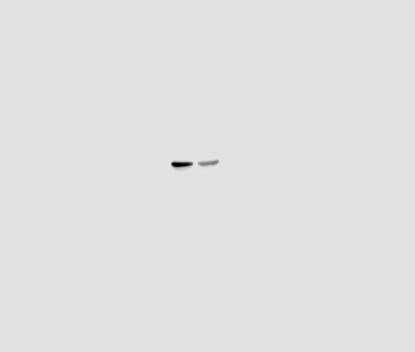 | 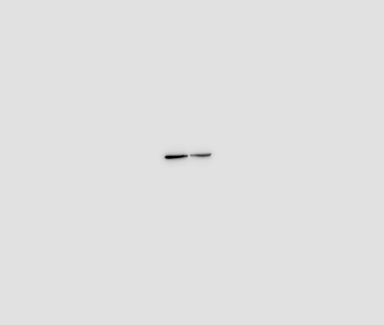 |
| **NSUN2**  **(110 kd)** | 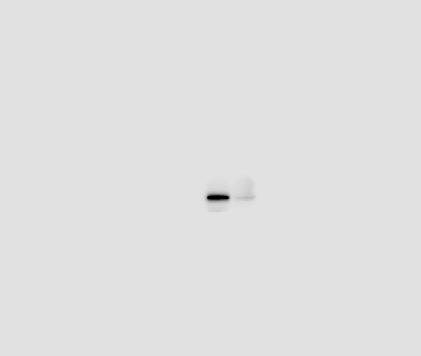 | 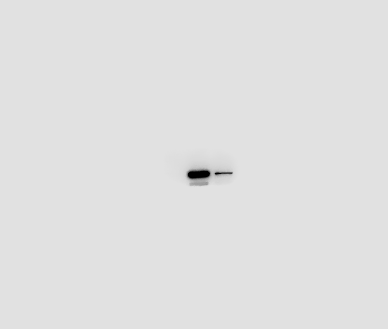 |
| **GAPDH**  **(37 kd)** | 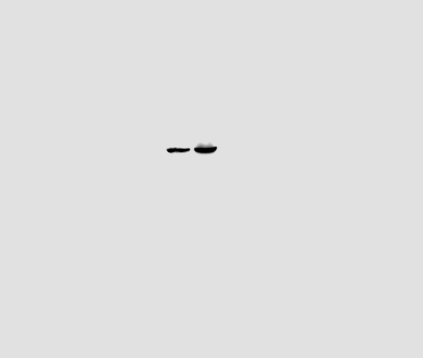 | 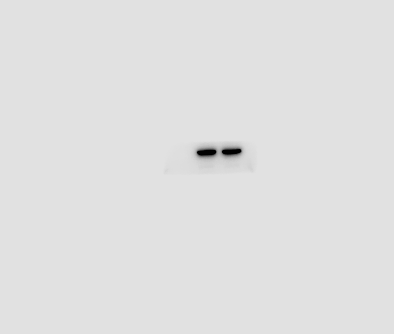 |

**Fig. 6D**

|  | **lane 1-lane 3:**  **MCF7-Ctrl, MCF7-NSUN2-WT, MCF7-NSUN2-DM** | **lane 1-lane 3:**  **T47D-Ctrl, T47D-NSUN2-WT, T47D-NSUN2-DM** |
| --- | --- | --- |
| **HGH1**  **(50 kd)** | 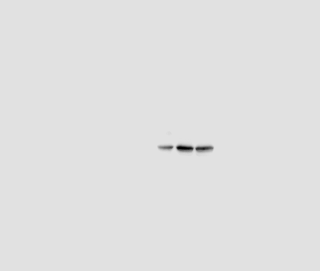 | 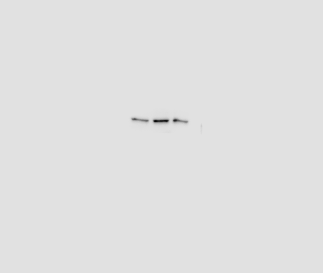 |
| **NSUN2**  **(110 kd)** | 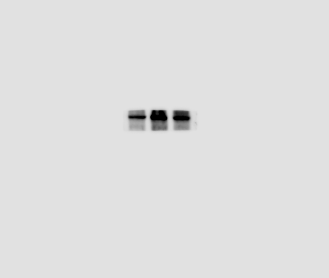 | 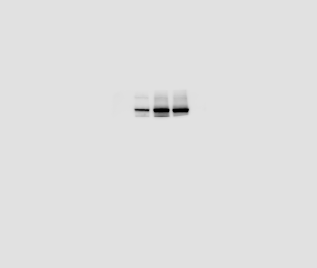 |
| **GAPDH**  **(37 kd)** | 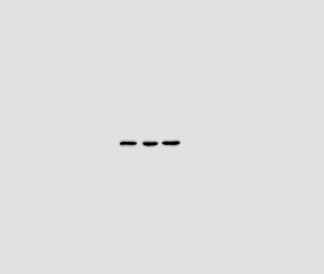 | 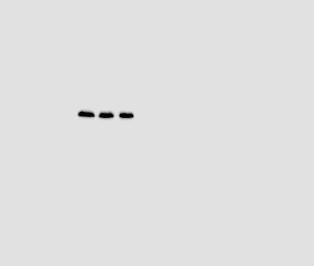 |

**Fig. 7E**

|  | **lane 1-lane 4: MCF7-siNC, MCF7-siYBX1; MCF7sgNC, MCF7sgYBX1(sgYBX1 transfection failed,** **please ignore)** | **lane 1-lane 4: T47D-siNC, T47D-siYBX1; T47DsgNC, T47DsgYBX1(sgYBX1 transfection failed,** **please ignore)** |
| --- | --- | --- |
| **HGH1**  **(50 kd)** | 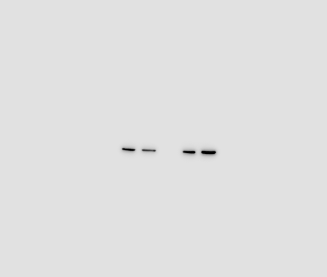 | 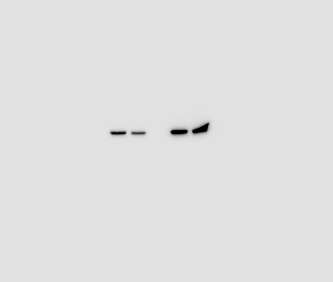 |
| **YBX1**  **(55 kd)** | 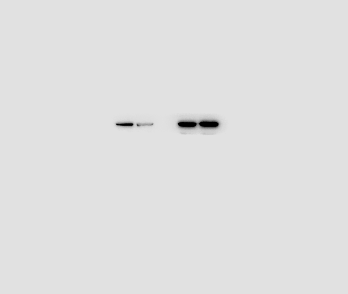 | 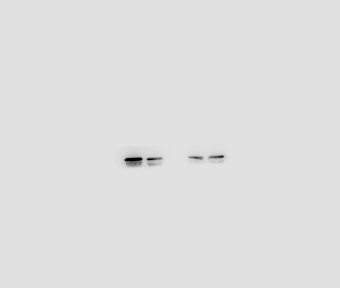 |
| **GAPDH**  **(37 kd)** | 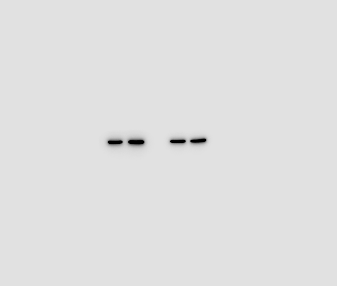 | 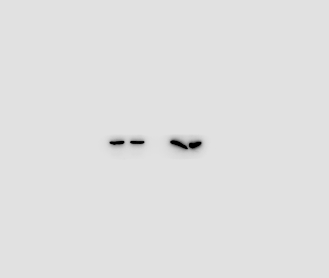 |

**Fig. 7F**

|  | **lane 1-lane 3:**  **MCF7-Ctrl, MCF7-YBX1-WT, MCF7-YBX1-Mut** | **lane 1-lane 3:**  **T47D-Ctrl, T47D-YBX1-WT, T47D-YBX1-Mut** |
| --- | --- | --- |
| **HGH1**  **(50 kd)** | 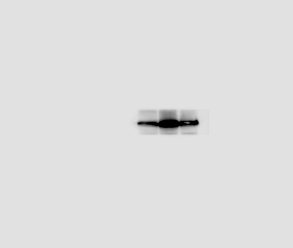 | 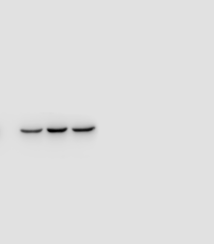 |
| **YBX1**  **(55 kd)** | 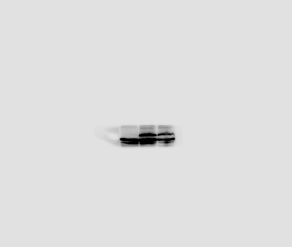 | 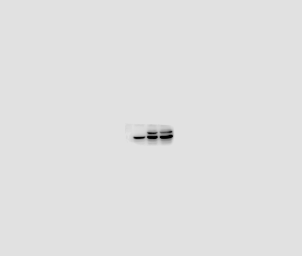 |
| **GAPDH**  **(37 kd)** | 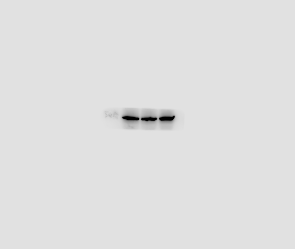 | 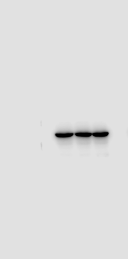 |

**Fig. 7H**

|  | **lane 1-lane 8: MCF7-siNC, MCF7-NSUN2-siNC, MCF7-NSUN2-siYBX1, MCF7-siYBX1；MCF7-siNC, MCF7-NSUN2-siNC, MCF7-NSUN2-siYBX1, MCF7-siYBX1；** | **lane 1-lane 4: T47D-siNC, T47D -NSUN2-siNC, T47D -NSUN2-siYBX1, T47D -siYBX1** |
| --- | --- | --- |
| **HGH1**  **(50 kd)** | 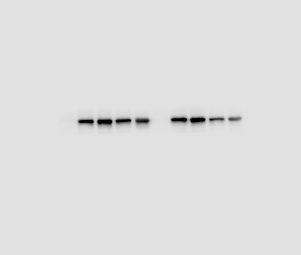 | 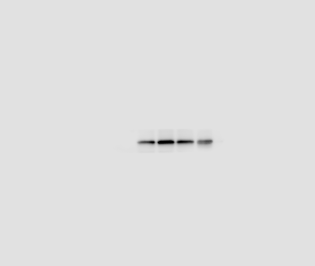 |
| **YBX1**  **(55 kd)** | 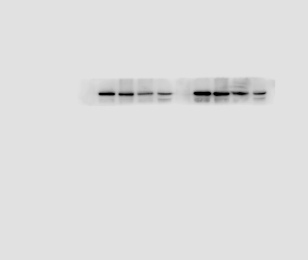 | 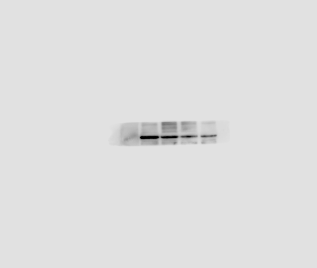 |
| **NSUN2**  **(110 kd)** | 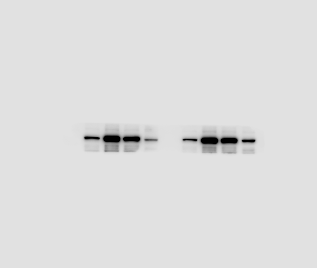 | 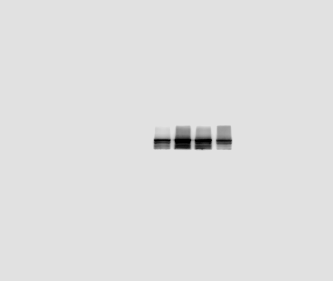 |
| **GAPDH**  **(37 kd)** | 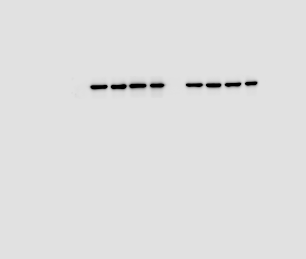 | 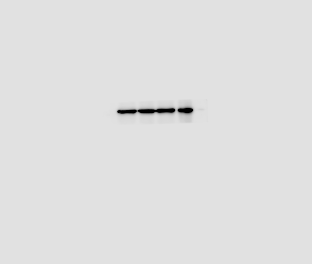 |

**Fig.S1E**

|  | **lane 1-lane 8:**  **MCF7shNC, MCF7shNSUN2, MCF7shNSUN2-oeNSUN2-WT, MCF7shNSUN2-oeNSUN2-DM; MCF7shNC, MCF7shNSUN2, MCF7shNSUN2-oeNSUN2-WT, MCF7shNSUN2-oeNSUN2-DM** |
| --- | --- |
| **NSUN2**  **(110 kd)** | 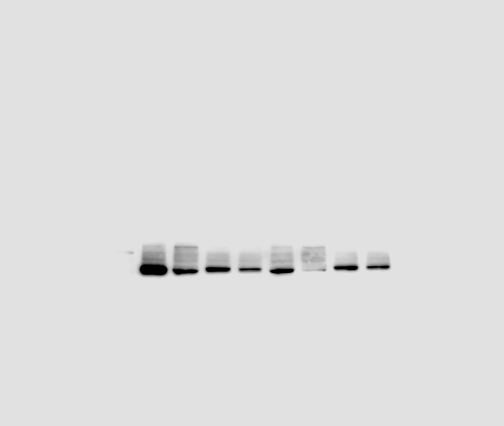 |
| **GAPDH**  **(37 kd)** | 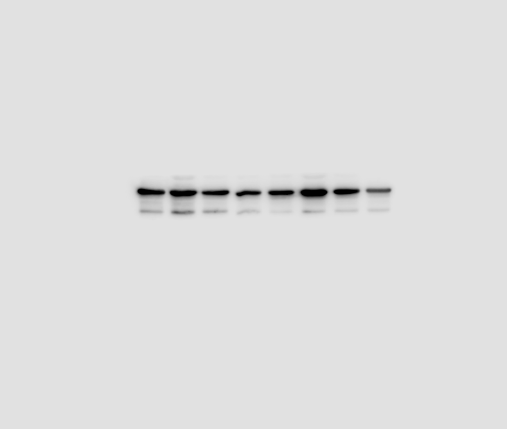 |

**Fig.S1F**

|  | **lane 1-lane 6:**  **MCF7-siNC, MCF7-siNSUN2-1, MCF7-siNSUN2-2, T47D-siNC, T47D-siNSUN2-1, T47D-siNSUN2-2** |
| --- | --- |
| **NSUN2**  **(110 kd)** | 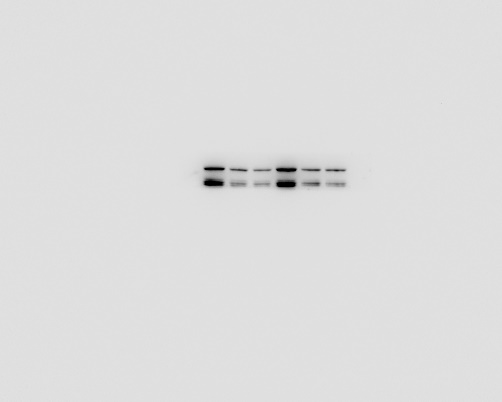 |
| **GAPDH**  **(37 kd)** | 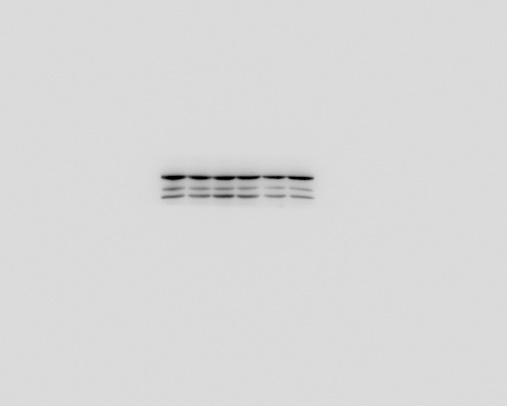 |

**Fig.S1G**

|  | **lane 1-lane 3: MCF7shNC, MCF7oeNSUN2-WT, MCF7oeNSUN2-DM** | **lane 1-lane 3: T47DshNC, T47DoeNSUN2-WT, T47DoeNSUN2-DM** |
| --- | --- | --- |
| **NSUN2**  **(110 kd)** | 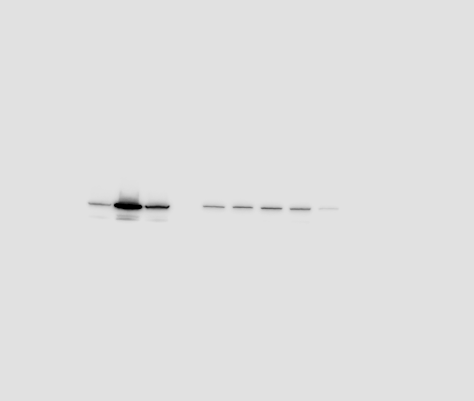 | 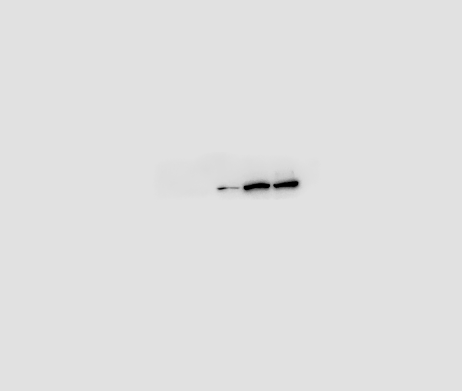 |
| **GAPDH**  **(37 kd)** | 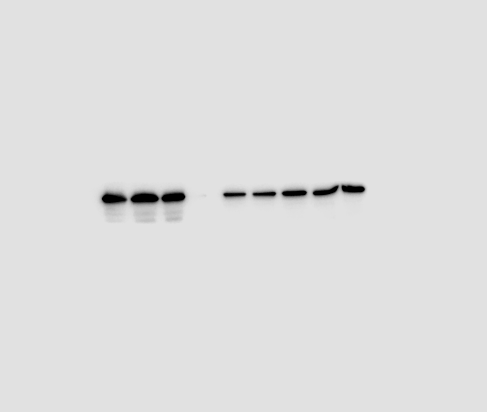 | 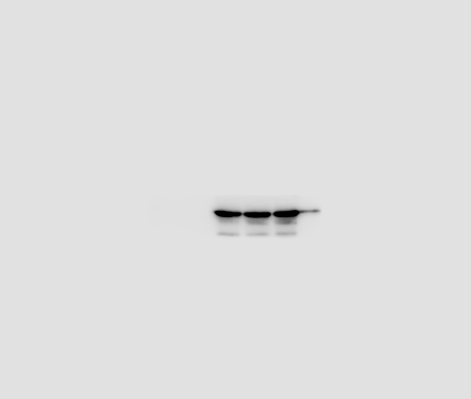 |

**Fig.S1H**

|  | **lane 1-lane 4: MCF7shNC, MCF7shNSUN2, T47DshNC, T47DshNSUN2** |
| --- | --- |
| **NSUN2**  **(110 kd)** | 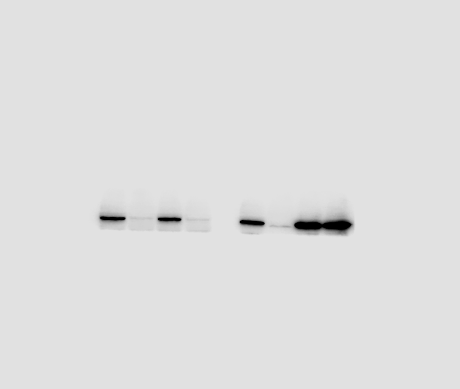 |
| **GAPDH**  **(37 kd)** | 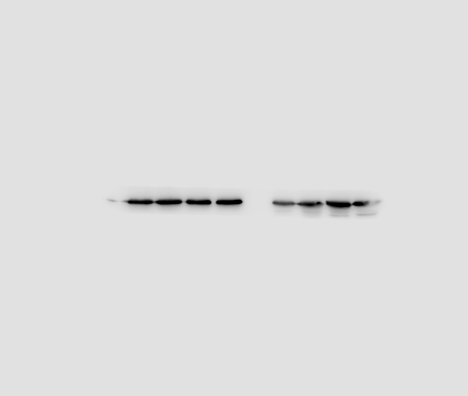 |

**Fig.S2C**

|  | **lane 6-lane 7: MCF7-siNC, MCF7-siHGH1** | **lane 1-lane 2: T47D-siNC, T47D-siHGH1** |
| --- | --- | --- |
| **HGH1**  **(50 kd)** | 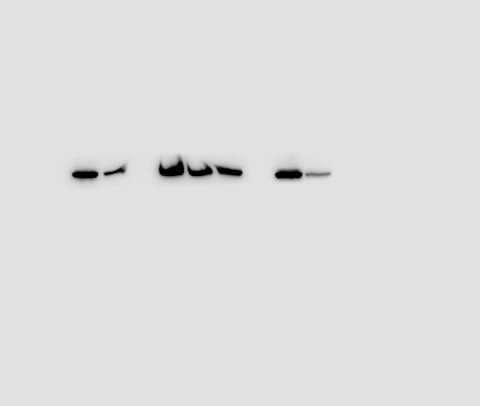 | 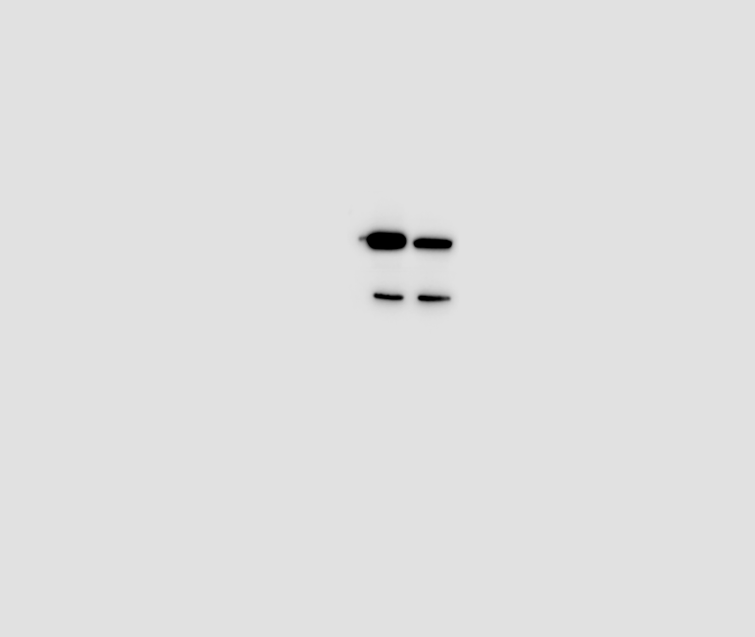 |
| **GAPDH**  **(37 kd)** | 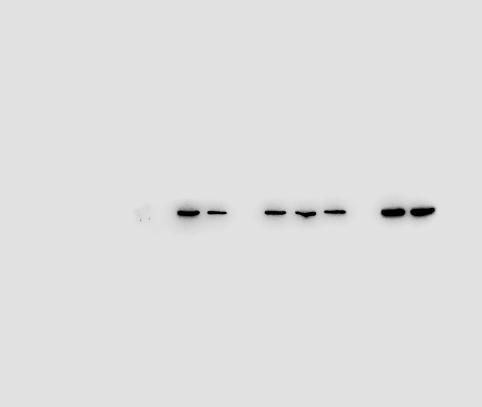 |  |

**Fig.S2D**

|  | **lane 1-lane 2: MCF7-Ctrl, MCF7-oeHGH1** | **lane 1-lane 6: T47D-Ctrl, T47D-oeHGH1;**  **T47D-Ctrl, T47D-oeHGH1; T47D-Ctrl, T47D-oeHGH1;** |
| --- | --- | --- |
| **HGH1**  **(50 kd)** | 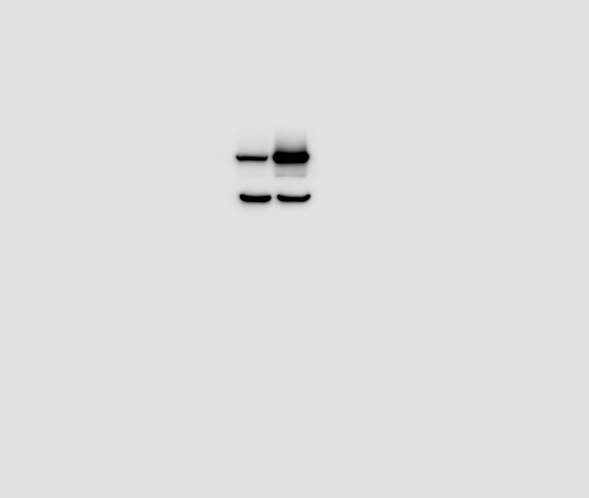 | 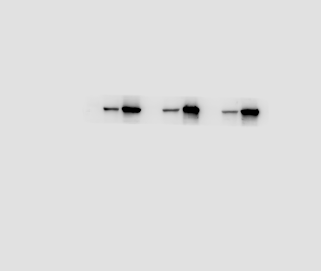 |
| **GAPDH**  **(37 kd)** |  | 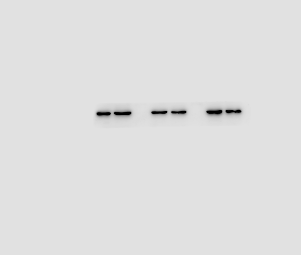 |

**Fig.S2F**

|  | **lane 1-lane 4: MCF7-shNC, MCF7-shHGH1, T47D-shNC, T47D-shHGH1** |
| --- | --- |
| **HGH1**  **(50 kd)** | 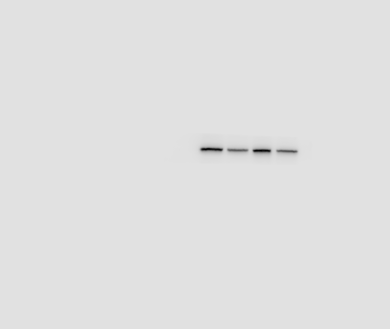 |
| **GAPDH**  **(37 kd)** | 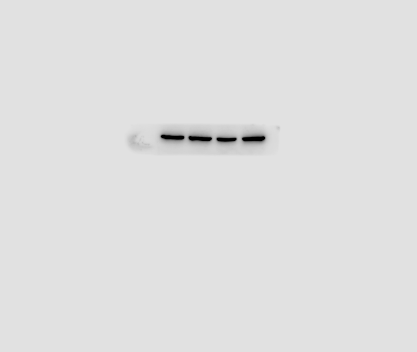 |

**Fig.S2H**

|  | **lane 1-lane 3: MCF7-shNC, MCF7-shHGH1, MCF7-shNSUN2oeHGH1** | **lane 1-lane 6: T47D-shNC, T47D-shHGH1, T47D-shNSUN2oeHGH1; T47D-shNC, T47D-shHGH1, T47D-shNSUN2oeHGH1;** |
| --- | --- | --- |
| **NSUN2**  **(110 kd)** | 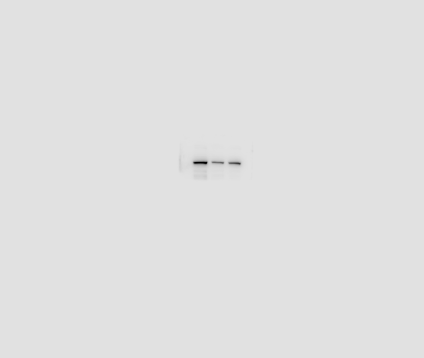 | 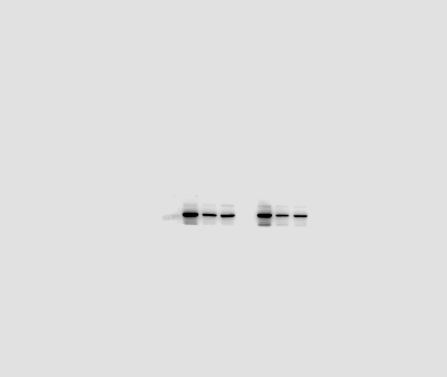 |
| **HGH1**  **(50 kd)** | 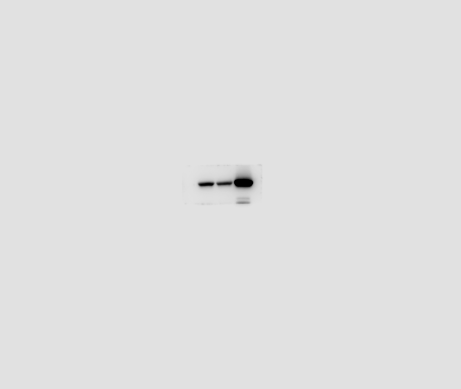 | 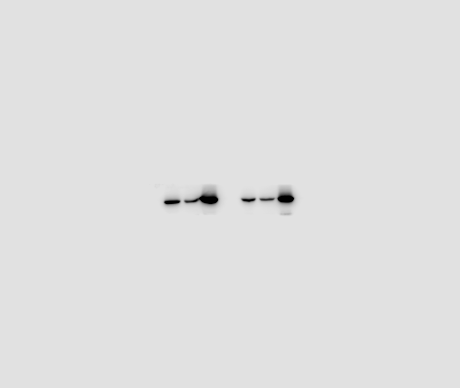 |
| **GAPDH**  **(37 kd)** | 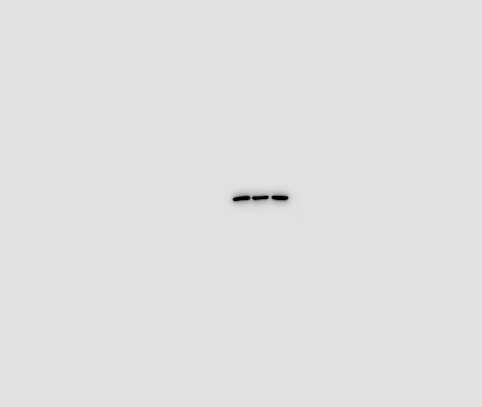 | 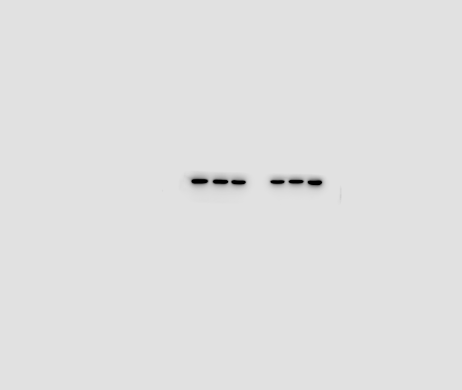 |

**Fig.S3B**

|  | **lane 1-lane 4: MCF7-shNC, MCF7-shNSUN2, T47D-shNC, T47D-shNSUN2** |
| --- | --- |
| **NSUN2**  **(110 kd)** | 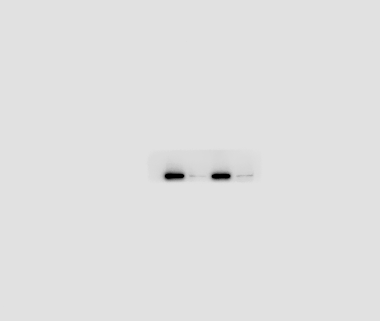 |
| **HGH1**  **(50 kd)** | 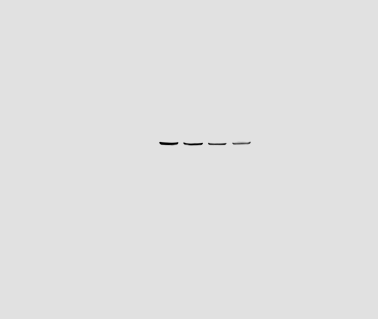 |
| **GAPDH**  **(37 kd)** | 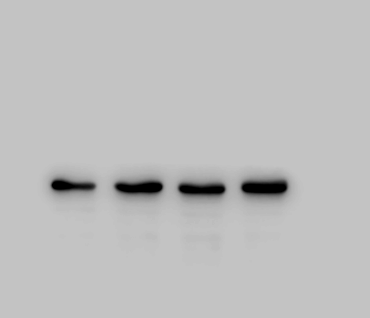 |

**Fig.S4B**

|  | **lane 1-lane 6: MCF7-siNC, MCF7-siNSUN2-1, MCF7-siNSUN2-2,**  **T47D-siNC, T47D-siNSUN2-1，T47D-siNSUN2-2** |
| --- | --- |
| **Anti-puro** | 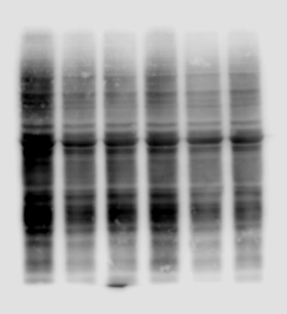 |
| **NSUN2**  **(110 kd)** | 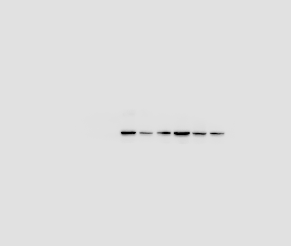 |
| **GAPDH**  **(37 kd)** | 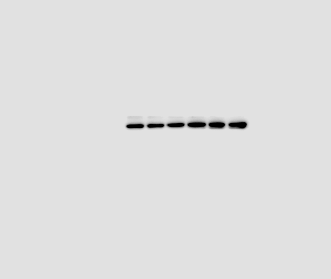 |

**Fig.S4C**

|  | **lane 1-lane 2: MCF7-siNC, MCF7-siYBX1** | **lane 1-lane 2:T47D-siNC, T47D-siYBX** |
| --- | --- | --- |
| **Anti-puro** | 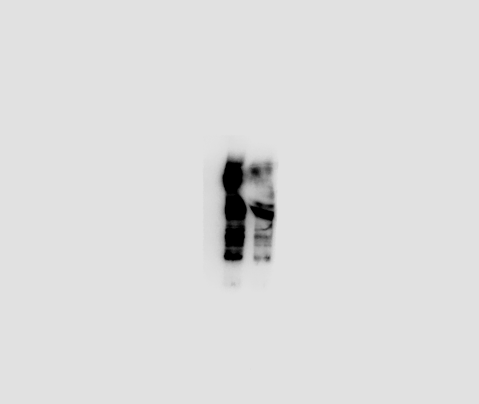 | 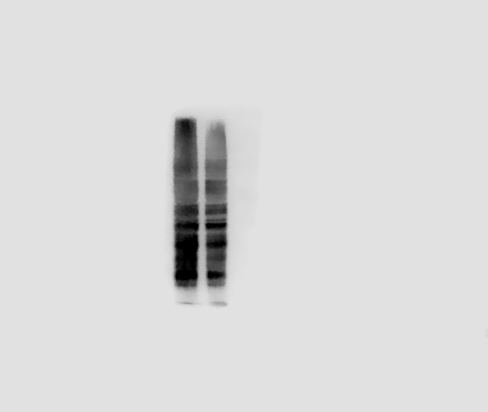 |
| **YBX1**  **(55 kd)** | 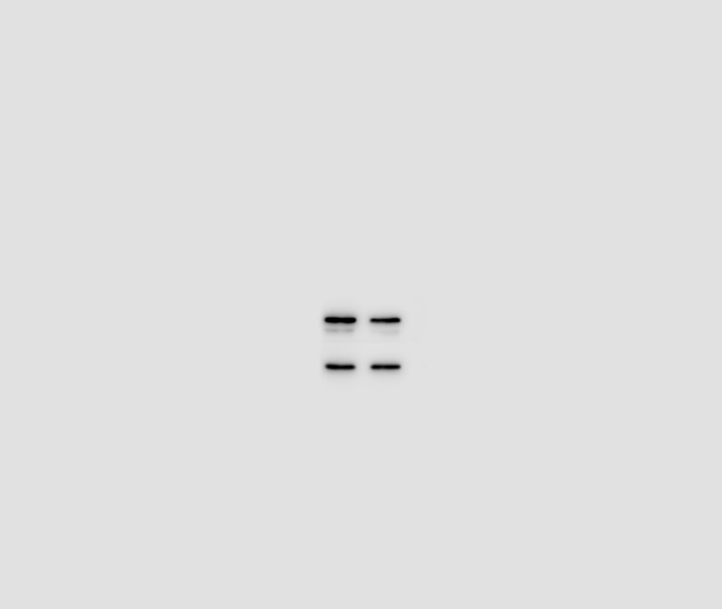 | 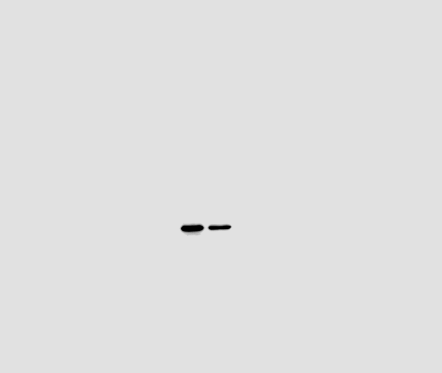 |
| **GAPDH**  **(37 kd)** |  | 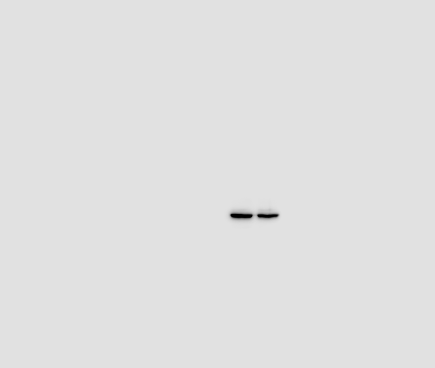 |
