# Supplementary material for: NSUN2/YBX1 promotes the progression of breast cancer by enhancing HGH1 mRNA stability through m5C methylation
Source: Breast Cancer Res. 2024 Jun 6;26:94. doi: 10.1186/s13058-024-01847-0 (PMC11155144; doi:10.1186/s13058-024-01847-0)
Supplement: Supplementary file 7 — Supplementary Material 7 [file 13058_2024_1847_MOESM7_ESM.docx]

**Fig.S1| NSUN2 has a positive effect on breast cancer.**

**(A)**-**(C)** H-score show the differential expression of NSUN2 in BC tested by IHC. **(D)** BALB/c nude mice (n=5 per group) were surgical injected into the mammary fat pads with MCF7-shNC, MCF7-shNSUN2 or MCF7-shNSUN2oeNSUN2 cells. **(E)** The MCF7 cells were transfected with shNSUN2, and the oeNSUN2 lentivirus was introduced into the MCF7-shNSUN2 cells. The expression levels were subsequently confirmed by western blot. **(F)** MCF7 and T47D cells were transfected with siNSUN2 or siNC for 48 hours, and subsequently, whole cell lysates were analyzed by western blot using the appropriate antibodies. **(G)** MCF7 and T47D cells were stably transfected with NSUN2-WT or NSUN2-DM lentivirus, and **(H)** stably transfected with shNSUN2. **p≤0.01, ***p≤0.001, ****p≤0.0001 by t test.

**Fig.S2| Suppression of HGH1 delays BC progression.**

**(A)-(F)** RT-qPCR and western blot clarified the HGH1 expression of MCF7 and T47D cells after transfected with siHGH1 or oeHGH1 and shHGH1 lentivirus. **(G)** BALB/c nude mice (n=5 per group) were surgical injected into the mammary fat pads with MCF7-shNC, MCF7-shHGH1 or MCF7-shNSUN2oeHGH1 cells. **(H**) MCF7 and T47D cells pre-treated with shNSUN2 were transfected with LV-HGH1 lentivirus and western blot analysis of HGH1 and NSUN2 expression. Data are mean ± SD of three independent experiments. **p≤0.01, ****p≤0.0001 by t test.

**Fig.S3| NSUN2 regulates the expression of HGH1.**

**(A)** and **(B)** RT-qPCR and Western blot assays tested the expression of HGH1 in BC cell lines after NSUN2 knockdown. **(C)** RT-qPCR assays verified NSUN2 and HGH1 expression levels in MCF7 and T47D cells. ns. no significant; Data are mean ± SD of three independent experiments. **p≤0.01, ***p≤0.001, ****p≤0.0001 by t test.

**Fig.S4| Inhibiting NSUN2 or YBX1 reduces protein synthesis in BC cells.**

**(A)** Nascent Protein level was detected by Alexa Fluor 594 staining where the alkyne-modified protein utilizes a chemoselective ligation or click reaction between an azide and alkyne. The FITC staining corresponds to immunofluorescence assay with anti-NSUN2 antibody and secondary antibody with FITC. Hoechst staining indicates the position of the nucleus due to its ability to bind to DNA. **(B)** and **(C)** MCF7 and T47D cells transfected with siNSUN2 (B) or siYBX1 (C) were treated with 1μM puromycin for 1h and the whole cell lysates were detected by western blot using an anti-puromycin antibody.

**Fig.S5| Knockdown of YBX1 reduces HGH1 expression and BC progression.**

**(A)** Analyzing protein expression differences by calculating grayscale values of western blot assays. **(B)** Cellular proliferation rates of BC cells with YBX1-Knockdown by CCK-8 assays. **(C)** FITC and PE fluorescence dyes were used to detect the cell apoptosis change by flow cytometry after YBX1 Knockdown. **(D)** PI fluorescence dye was used to detect the cell cycle alteration by flow cytometry upon YBX1 Knockdown. ns. no significant; Data are mean ± SD of three independent experiments. *p≤0.05, **p≤0.01, ****p≤0.0001 by t test.
